# Inorganic Hole Transport Materials for Advancing *n‐i‐p* Perovskite Solar Cells: A Comprehensive Review

**DOI:** 10.1002/smll.74010

**Published:** 2026-05-31

**Authors:** Maham Akhlaq, Hongxia Wang, Tuquabo Tesfamichael

**Affiliations:** ^1^ School of Mechanical Medical and Process Engineering Faculty of Engineering Queensland University of Technology Brisbane Queensland Australia; ^2^ Centre for Materials Science Faculty of Science Queensland University of Technology Brisbane Queensland Australia; ^3^ School of Chemistry and Physics Faculty of Science Queensland University of Technology Brisbane Queensland Australia; ^4^ Centre for Biomedical Technology Faculty of Science Queensland University of Technology Brisbane Queensland Australia

**Keywords:** hole transport layer, inorganic materials, metal oxide, perovskite solar cells, power conversion efficiency

## Abstract

The rapid advancement of perovskite solar cells (PSCs) through high‐power conversion efficiencies (PCEs) and low fabrication costs made them a potential candidate for the next generation of photovoltaic technology. Although, the inverted (*p‐i‐n)* configuration of the PSCs has recently gained attention due to low temperature production, the regular (*n‐i‐p)* architecture remains a benchmark model for inorganic HTLs due to high PCEs and well characterized interfacial energy levels. Hole transport layer plays a vital role in extracting photogenerated holes and minimizing charge recombination and energy losses in *n*‐*i*‐*p* architecture. While organic materials like spiro‐OMeTAD have dominated HTL research, their limitations in stability, cost, environmental sustainability, and scalability have steered interest in inorganic alternatives. This comprehensive review systematically explores recently progress in inorganic HTLs for *n*‐*i*‐*p* PSCs, focusing on metal oxides, metal chalcogenides and emerging inorganic compounds. Important aspects of the HTLs required for enhancing the PSCs, including optical properties, energy gap, band alignment, deposition techniques and interfacial engineering strategies with emphasis on their influence on PCEs, stability and commercial viability is carried out. By consolidating recent advancements and identifying remaining key challenges, this review offers a critical foundation for advancing the design and optimization of efficient, stable and scalable n‐i‐p PSCs.

## Graphical abstract

Inorganic hole transport materials are comprehensively reviewed for advanced n‐i‐p perovskite solar cells through improved photovoltaic parameters, reduced ion migration and enhanced operational stability. Metal oxides, chalcogenides and hybrid inorganic‐organic hole transport layers demonstrate strong potential for achieving efficient, durable and scalable perovskite solar cells using solution processing and vacuum deposition techniques.

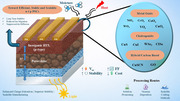

## 1 Introduction

**FIGURE 1:**
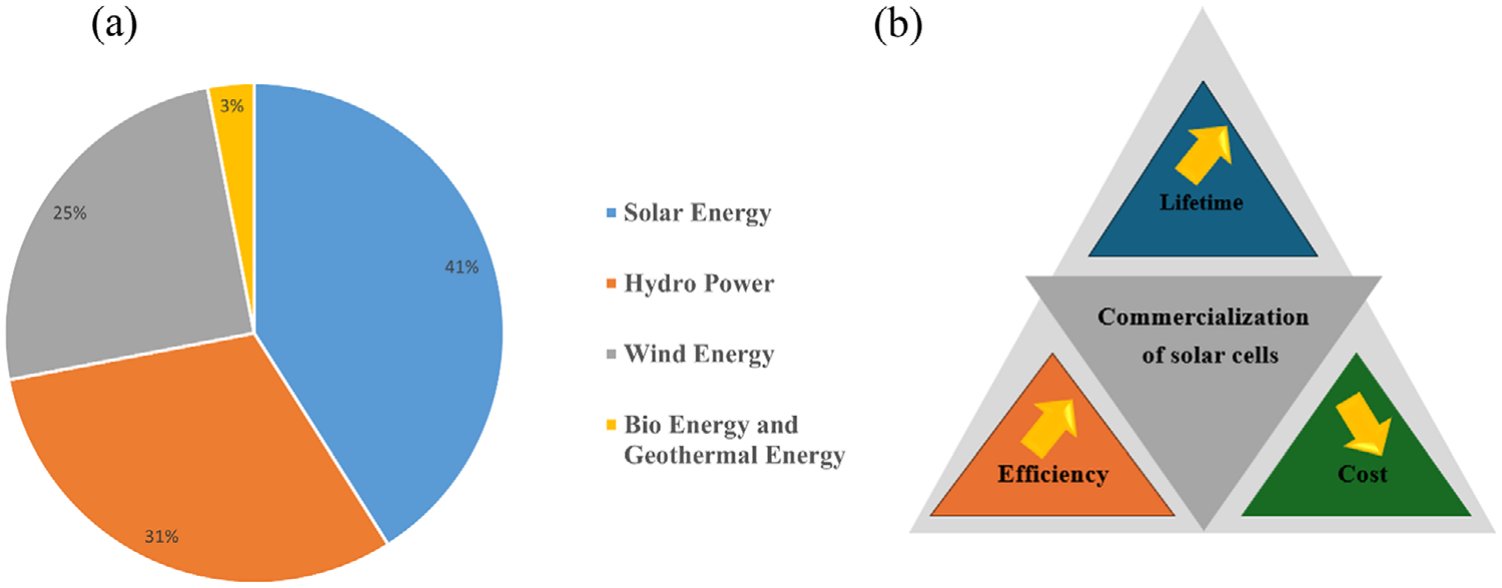
(a) Current (2023) global renewable energy generation [2] (b) The golden triangle for the commercialization of the solar cells.

Energy consumption across the globe is steadily rising, and this increases demand for additional energy resources in the global energy sector. The rapid depletion of fossil fuels and their environmental impact have promoted the research community to develop sustainable and clean energy alternatives [1]. Out of the 9.7 TWH global total energy generated in 2023, 46.4% (about 4.5 TWH) was obtained from clean energy resources [2, 3]. These clean energy resources generally include solar, wind, hydropower, biomass, and geothermal. Within the category of these clean energy resources, solar energy stands out as a leading and innovative renewable energy solution with a significantly large capacity of 41% as shown in Figure 1a [2]. Solar energy is the most sustainable energy resource with abundant energy entering the earth every day, and it can be harvested in small‐ and large‐scale production effectively. It has several advantages over the other alternative energy resources, such as wind, as it has little disturbance (e.g. noise, space, safety, etc) on the community.

Currently, solar energy only accounts for nearly 19.2% of the global total energy production [2, 4]. This low contribution is attributed to the higher cost, lower efficiency, requirements of high‐tech, and lower manufacturing throughput. Researchers across the globe are working on the “golden triangle” shown in Figure 1b to overcome these limitations through innovative solar cells and engineering of the material chemistry and re‐design of the existing photovoltaics (PVs) for commercialization of efficient, durable, and cost‐effective solar cells [5, 6, 7].

Photovoltaics (PVs) are generally classified based on their working principles and the type of absorbing semiconductor materials used [8, 9]. Mono‐ and poly‐ crystalline silicon solar cells with a complex fabrication process, high cost for installation and transportation, and a significant amount of material usage are currently dominating the market with the best efficiency of 27.6% [10]. Thin film solar cells such as Copper indium gallium selenide (CIGS) and Cadmium Telluride (CdTe) may overshadow the high production cost and extensive material usage in silicon solar cells, but their best reported lab efficiency is relatively lower: 23.6% (CIGS), 23.1% (CdTe) and 15.8% (CZTS) [10, 11]. On the other hand, scientists and engineers are currently researching on emerging types of solar cells, including organic solar cells (19.2%), dye sensitized solar cells (13%), quantum dots solar cells (19.1%) and perovskite solar cells (27%) [12]. Among these emerging solar cells, perovskite solar cells have shown great potential with a growing performance in a short period of time (since 2009) to overcome the issue of efficiency and cost [13]. This article reviews perovskite solar cells with a prime focus on inorganic hole transport materials (HTM) in n‐i‐p perovskite solar cells (PSCs).

**FIGURE 2:**
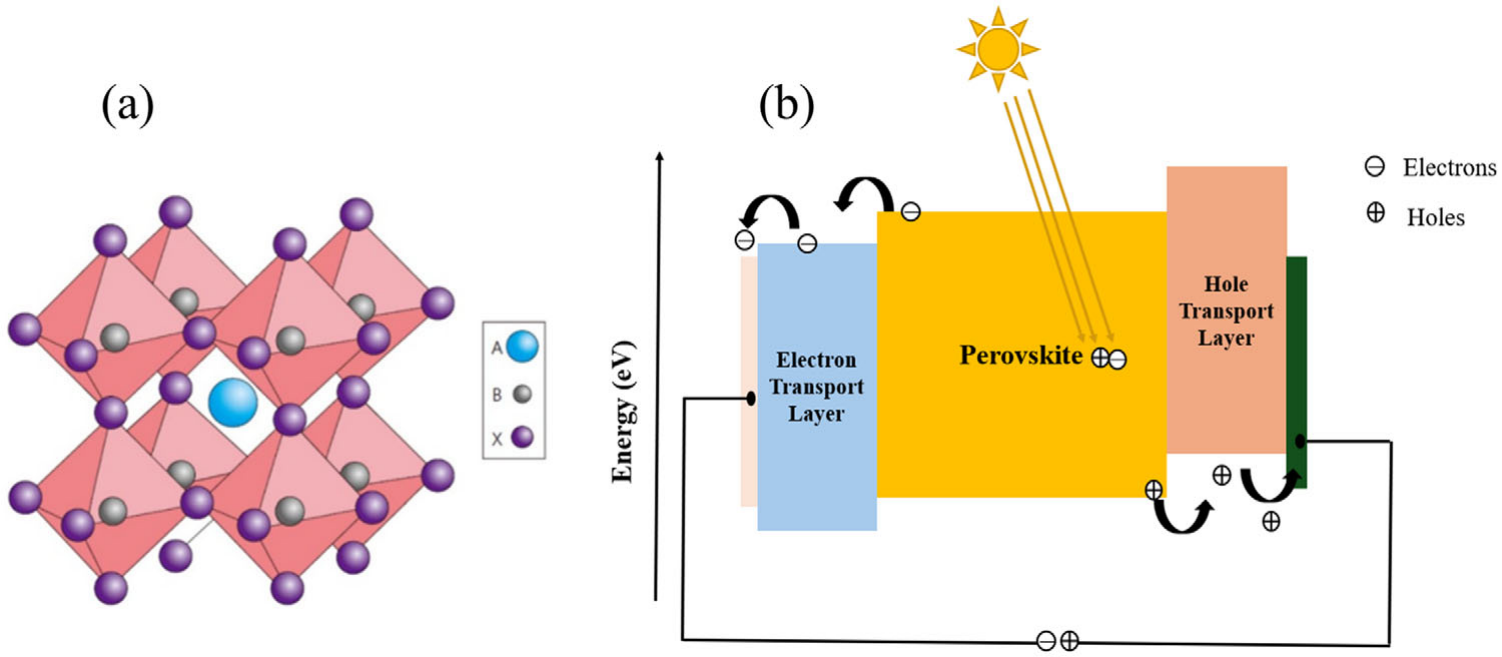
(a) Cubic perovskite crystal structure with chemical formula ABX_3_ [19] (b) Energy level and charge transfer of ETL and HTL of a PSC.

Perovskite materials have gain considerable properties because of their excellent light absorption coefficient, high electron and hole mobility, tuneable bandgap for broader light absorption, low temperature processing, large diffusion length and lower defect density [14, 15, 16, 17]. With around 27% certified world‐record efficiency achieved today, PSCs have shown a competitive potential for sustainable photovoltaics (PV) technology [10, 18]. Perovskites are materials with a chemical formula ABX_3_, where A and B are cations of variable sizes, and X is an anion [19]. As shown in Figure 2a, the crystal structure of perovskite consists of a cubo octahedral structure, which is shared with twelve other anions. Also the cation provide the stability by sharing the sides with six anions [20]. The perovskites are classified into oxide perovskites (X as oxygen) and halide perovskites (X as halogon). Oxide‐based perovskites show excellent electrical properties in superconductivity and ferroelectricity, whereas halide perovskites are considered as excellent light‐absorbing materials owing to their semiconducting property, structural and compositional tunability, and excellent quantum yield for photoluminescence [21, 22]. In halide‐based ABX_3_ perovskite, A represents methylammonium, formamidinium or cesium, B as a metal cation (Lead or Tin) and X as a halogen anion (Cl, Br or I) [23].

Generally, PSC structure (Figure 2b) consists of an absorber layer (example: CH_3_NH_3_PbX_3_), which is inserted between the two charge transports layers; electron transport (n‐type) and hole transport (p‐type) layers. The photo‐generated charges are extracted by an external circuit through the current collectors that are in contact with the ELT or HTL.

When the light falls on the perovskite‐absorber, an electron‐hole pair is created, and these charges are separated by the n‐type and p‐type carrier‐transporting materials to generate free charge carriers. Electron reaches the external circuit, and the oxidation state of the perovskite is restored by the hole transport layer. The amount of photo‐generated current depends on several factors, such as electrical, electronic, optical, and chemical properties of perovskite and charge extracting materials, as well as the thickness, energy band, interfaces, and quality of the perovskite and the charge transport layers [24, 25].

**FIGURE 3:**
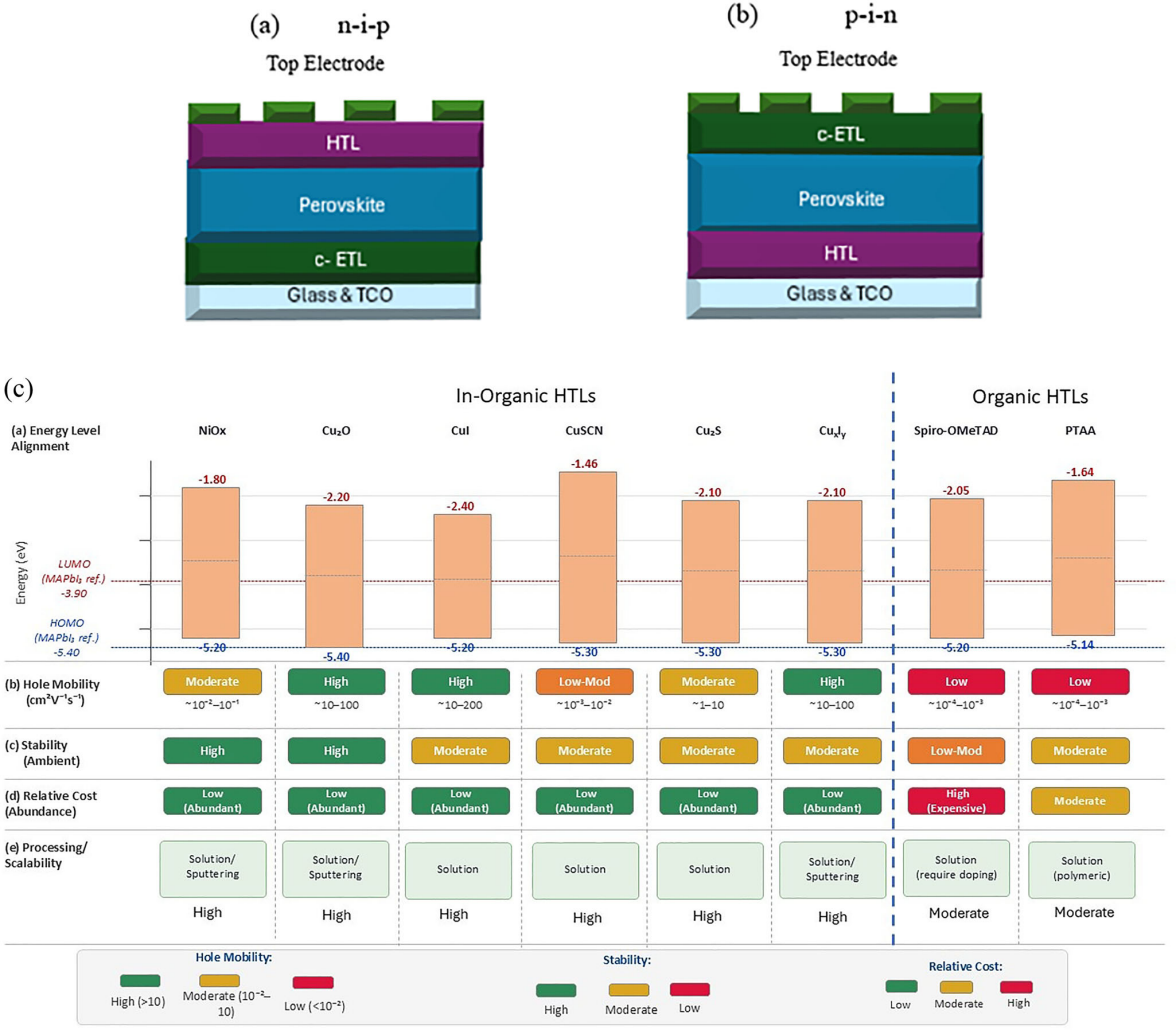
PSC structures (a) n‐i‐p (regular structure), and (b) p‐i‐n (inverted structure). (c) Comparative overview of representative hole transport materials for n–i–p perovskite solar cells, including energy level alignment, hole mobility, ambient stability, and relative cost. Values are compiled from literature and represent typical ranges; stability classifications depend on testing conditions and encapsulation [32, 33, 34, 35].

The mainstream device structures of the PSCs are shown in Figure 3, including comprehensive integrated energy levels alignment, hole mobility, stability and cost analysis of the solar cells. The structures are classified based on the charge transport layer position, n‐i‐p (regular) or p‐i‐n (inverted). The position of the charge transport layer subsequently impacts the power conversion efficiency (PCE) of the solar cell [26, 27], mainly due to the interface charge collection, energy alignment and need of protection barrier from the external environment [28, 29]. For the charge transport layers, the occupied molecular orbital energy levels must be compatible with the perovskite absorber to avoid recombination of charges and degradation of the perovskite layer. Within the PSCs, the hole transport layer (HTL) plays a crucial role in determining the efficiency and stability of the solar cell, including the recombination kinetics, charge extraction efficiency, energy level alignment and charge transfer time [30, 31]. For example, if miss alignment of the energy level between the perovskite and hole transport layer occurred, the holes will not be efficiently extracted, resulting in either charge accumulation and/or an increase in trap‐assisted recombination at the interface that can generate undesirable heat and reaction of radicals across the Perovskite/HTL interface.

Figure 3c shows a comparative overview of the energy levels, hole mobility, stability, relative cost and processing/scalability of commonly used hole transport organic and inorganic materials for PSCs. For efficient hole extraction, the valence band maximum (VBM) of the perovskite must be compatible with the highest occupied molecular orbital (HOMO) of the valence band of the HTL [36]. The HOMO represents the energy level from which electrons can be easily extracted, while the lowest unoccupied molecular orbital (LUMO) represents the energy level where electrons can be accepted. The primary function of HTL is to have high hole mobility for extract the holes generated in the perovskite layer and transport them toward the electrode while effectively reducing recombination by preventing electrons from reaching this electrode.

Beyond band alignment, the HTLs key parameters includes stability and cost. As shown in Figure 3c, NiO_x_ and copper‐based inorganic HTL have favorable stability and cost effectiveness over organic HTLs.

Based on the main characteristics of the hole transport layer shown in Figure 3c, the materials should possess:

- High hole mobility and carrier concentration to ensure fast and efficient charge transport [37].
- Chemically stable material, such as inorganic metal oxides, to enhance device life.
- Have an energy level that can be aligned with the perovskite absorbing layer to minimize energy barriers. Aligned VBM of the perovskite with the HTL will reduce charge recombination and improve the charge transport [38].
- Chemically compatibility with the perovskite absorber, the metal electrode, and/or any interfacial adjacent layer to avoid unwanted reactions and degradation [39, 40, 41].
- Nanostructured surface with hydrophobic properties to increase device stability.
- Higher transparency to reduce the absorption screen effect in p‐i‐n PSCs [42].

HTLs are generally classified as inorganic and organic materials, and each material has its own benefits and limitations. In last years, several review articles have discussed HTL materials for perovskite solar cells with a focus on organic or hybrid systems in both the architectures of perovskite solar cells. Reviews published in recent years rarely focuses on inorganic HTL tailored for *n‐i‐p* devices in terms of method and efficiency, where the interfacial energetics and working mechanics differs fundamentally from *p‐i‐n* structure. This review paper focuses primarily on inorganic HTLs for the *n‐i‐p* architecture, which are sufficiently investigated in device level to enable meaningful comparison in terms of performance, stability and processing compatibility in future PSCs. Emerging materials with limited device‐level validation are discussed separately to provide a forward‐looking perspective of the HTLs in n‐i‐p PSCs.

## 2 Organic HTL for PSCs

For the p‐i‐n structure with Pb‐based perovskite, a PCE of 26.7% (certified as 26.09%) has been reported, while the performance of PSCs in the n‐i‐p structure still lags [43, 44]. By gaining a deeper understanding of energy losses in the n–i–p structure, it would be possible to achieve the highest thermodynamic efficiency limit of the PSCs, which is about 31.0% [45, 46].

In the n–i–p devices, the device efficiency and stability largely depend on the HTL material and their charge transport properties at the HTL/perovskite interface [47]. The selection of the solvent for HTL deposition impacts the perovskite layer. Materials with enhanced charge transport properties exhibit high hole mobility, better hole extraction with reduced charge recombination, lower series resistance and reduced interfacial degradation. The performance of n‐i‐p is limited by their larger (E_g_–V_OC_) losses, typically exceeding 0.5 V and lower fill factors (FFs), often below 0.7. Reversing the configuration of perovskite solar cells from n‐i‐p to p‐i‐n (see Figure 3), leads to notable changes in device performance [48]. The underlying interlayer significantly influences the crystallization of perovskite layers, while the upper interlayer plays a critical role in passivating surface defects and shielding the absorber layer from harmful environmental conditions [49]. As a result, not all HTL materials are suitable for both configurations of the PSCs.

Today, the highest reported efficiency in the n‐i‐p PSC has been achieved using the small molecule 2,2′,7,7′‐tetrakis‐(N,N‐di‐4‐methoxyphenylamino)‐9,9′‐spirobifluorene(spiro‐MeOTAD) [50, 51, 52, 53]. With the LUMO with 2 eV higher than the conduction band maximum of perovskites, Spiro‐MeOTAD shows excellent electron blocking capabilities [54]. However, the HOMO between the perovskites and the Spiro‐MeOTAD lies in order of several meV, which results in voltage losses across the interface [55]. To reduce these issues and improve the hole mobility as well as the conductivity of the spiro‐MeOTAD, doping with other materials, including lithium salts and pyridine, was performed. These doping improve the efficiency and voltage losses but negatively affected the device stability [56]. Moreover, the high cost and hydroscopic nature of spiro‐MeOTAD urged researchers for alternative HTL materials. Another commonly used HTL material for n‐i‐p is poly(triarylamine) (PTAA) owing to its exceptional hole mobility (≈4 × 10^−3^ cm^2^ V^−1^ s^−1^) [57]. However, the high cost, volatile nature and requirement of doping to improve conductivity are some of the drawbacks of using PTAA as HTL [34, 58, 59].

## 3 Inorganic HTM for PSCs

Inorganic hole transport materials are emerging materials for use in PSCs due to their low cost, chemical and thermal robustness, and suitable electrical and electronic properties [60]. With high transparency, tunable energy levels, and tremendous stability against heat, moisture and light, semiconducting inorganic materials appear to have great potential as HTL. These materials have shown compatibility with the perovskite layer and protect the perovskite material from degradation [61]. Various inorganic materials, including oxides, sulphide, iodides as well as graphene derivatives, have been used for hole collection. Commonly known hole transport materials in the n‐i‐p architecture include oxides of copper and nickel, multifunctional oxides, graphene‐based materials, chalcogenide materials, and inorganic‐organic composites.

### 3.1 Copper‐Based HTL

Copper‐based materials including copper (I) oxide (Cu_2_O), Copper iodide (CuI), and copper sulphide (CuS)have been used as HTL in PSCs. These p‐type materials have improved energy levels, exceptional hole movement, enhanced conductivity, better optical properties, and good stability [62]. However, it should be noted that the stability improvements of many reported articles are based on short‐term, non‐standardized testing conditions and do not consistently distinguish between encapsulated and unencapsulated devices, limiting direct comparability. The photovoltaic parameters for various copper‐based HTL materials in n‐i‐p type PSCs are shown in Table 1.

**TABLE 1:** Copper‐based HTLs in PSCs.

Hole transport layer	Photoactive layer	Cell structure	Deposition method	V_oc_ (V)	J_sc_ (mA/cm^2^)	FF (%)	PCE (%)	Refs.
Cu_2_O	CH_3_NH_3_PbI_3_	FTO/TiO_2_/CH_3_NH_3_PbI_3_/Cu_2_O/Au	Spin‐coating	1.13	22.53	67.36	17.23	[67]
CuFeO_2_	Cs_0.10_(FA_0.4_MA_0.6_)_0.9_PbI_2.8_Br_0.2_	FTO/TiO_2_/ Cs_0.10_(FA_0.4_MA_0.6_)_0.9_PbI_2.8_Br_0.2_/CuFeO_2_/Au	Spin‐coating	1.01	23.6	66	15.6	[72]
CuS	CH_3_NH_3_PbI_3_	FTO/TiO_2_/ CH_3_NH_3_PbI_3_/CuS/Au	Spin‐coating	0.88	20.72	71	13.01	[77]
CuS	(FAPbI_3_)_0.78_(MAPbBr_3_)_0.14_(CsPbI_3_)_0.08_	FTO/TiO_2_/ (FAPbI_3_)_0.78_(MAPbBr_3_)_0.14_(CsPbI_3_)_0.08_/CuS/Au	Spin‐coating	0.75	21.23	74	11.72	[77]
Transfer printed CuI	MAFAPbBrI3	ITO/SnO_2_/MAFAPbBrI_3_/CuI/Au	Transfer printing	0.795	19.3	53.8	8.3	[91]
Undoped Spiro‐OMeTAD/Cu_9_S_5_	CH_3_NH_3_PbI_3_	ITO/N:SnO_2_/CH_3_NH_3_PbI_3_/ Undoped Spiro‐OMeTAD/Cu_9_S_5_/Au	Spin‐coating	1.05	22.32	73	17.10	[78]
Wurtzite – Cu_2_SnS_3_	Cs_0.05_(MA_0.17_‐FA_0.83_)_0.95_Pb(I_0.83_Br_0.17_)_3_	FTO/TiO_2_/Cs_0.05_(MA_0.17_‐FA_0.83_)_0.95_Pb(I_0.83_Br_0.17_)_3_/Wurtzite Cu_2_SnS_3_/Au	Spin‐coating	1.06	20.59	69	13.01	[84]
Zincblende – Cu_2_SnS_3_	Cs_0.05_(MA_0.17_‐FA_0.83_)_0.95_Pb(I_0.83_Br_0.17_)_3_	FTO/TiO_2_/Cs_0.05_(MA_0.17_‐FA_0.83_)_0.95_Pb(I_0.83_Br_0.17_)_3_/Zincblende Cu_2_SnS_3_/Au	Spin‐coating	1.01	13.03	61	7.87	[84]
Spiro‐OMeTAD/Cu_x_S	CH_3_NH_3_PbI_3_	FTO/SnO_2_/ CH_3_NH_3_PbI_3_/SpiroOMeTAD/Cu_x_S/Au	Thermal evaporation	1.125	23.10	71.50	18.58	[79]
Cu_2_O QDs	Cs_0.05_FA_0.81_MA_0.14_PbI_2.55_Br_0.45_	FTO/TiO_2_/Cs_0.05_FA_0.81_MA_0.14_PbI_2.55_Br_0.45_/Cu_2_O/Au	Spin‐coating	1.12	22.17	70.46	18.2	[68]
Cu_12_Sb_4_S_13_	CH_3_NH_3_PbI_3_	FTO/TiO_2_/ CH_3_NH_3_PbI_3_/ Cu_12_Sb_4_S_13_/Au	Spin‐coating	0.80	18.08	45	6.5	[82]
CuCrO_2_	Cs_0.05_(MA_0.15_FA_0.85_)_0.95_Pb(I_0.85_Br_0.15_)_3_	FTO/TiO_2_/Cs_0.05_(MA_0.15_FA_0.85_)_0.95_Pb(I_0.85_Br_0.15_)_3_/CuCrO_2_/Au	Spin‐coating	1.04	23.20	69	16.68	[71]
CuI/Cu	CH_3_NH_3_PbI_3_	FTO/TiO_2_/ CH_3_NH_3_PbI_3_/CuI/Cu/Au	Thermal Evaporation	0.85	22.99	47	9.24	[89]
CuInSe_2_	MAFAPb(I,Br,Cl)_3_	ITO/SnO_2_/ MAFAPb(I,Br,Cl)_3_/CuInSe_2_/Au	Spin‐coating	0.86	22.5	66	12.8	[92]
CuInGa(SSe)_2_	CH_3_NH_3_PbI_3_	FTO/TiO_2_/CH_3_NH_3_PbI_3_/CuInGa(SSe)_2_/Au	Spin‐coating	0.94	17.66	54.88	9.15	[93]
Spiro‐OMeTAD/Cu_2_O	CH_3_NH_3_PbI_3_	FTO/TiO_2_/ CH_3_NH_3_PbI_3_/Spiro‐OMeTAD/Cu_2_O/Ag	Spin‐coating/sputtering	1.03	22.46	74.1	17.11	[94]
rGO/CuI/rGO	PVSK	FTO/TiO_2_/PVSK/rGO/CuI/rGO/Au	Spin‐coating/thermal evaporation/spin‐coating	0.832	18.86	55	8.69	[90]
CuI/rGO	PVSK	FTO/TiO_2_/PVSK/CuI/rGO/Au	Thermal evaporation/spin‐coating	0.857	17.70	54.3	8.24	[90]
CuI	PVSK	FTO/TiO_2_/PVSK/CuI/Au	Thermal Evaporation	0.832	15.63	62	8.07	[90]
CuI	CH_3_NH_3_PbI_3−x_Cl_x_	FTO/m‐TiO_2_/CH_3_NH_3_PbI_3−x_Cl_x_/CuI/Au	Spray‐coating	0.61	22.3	42	5.8	[95]
CuI	CH_3_NH_3_PbI_3_	FTO/TiO_2_/CH_3_NH_3_PbI_3_/CuI/graphite	Doctor Blade	0.78	16.7	57	7.5	[96]
CuI	CH_3_NH_3_PbI_3_	FTO/TiO_2_/CH_3_NH_3_PbI_3_/CuI/Au	Thermal Evaporation	0.73	23.7	31	7.4	[97]
CuI	CH_3_NH_3_PbI_3_	ITO/TiO_2_/CH_3_NH_3_PbI_3_/CuI/Au	Spin‐coating	0.42	14.7	40	2.2	[98]
CuI_3−x_/CuI	CH_3_NH_3_PbI_3_	TiO_2_/CH_3_NH_3_PbI_3_/CuI_3−x_/CuI	Pressing	0.67	24.2	50	8.1	[99]
CuI	CH_3_NH_3_PbI_3_	FTO/TiO_2_/ CH_3_NH_3_PbI_3_/CuI/Au	Automated Drop‐Casting	0.55	17.8	62	6.0	[88]
Cu_2_ZnSnS_4_	CH_3_NH_3_PbI_3_	FTO/TiO_2_/CH_3_NH_3_PbI_3_/ Cu_2_ZnSnS_4_/Au	Spin‐coating	1.06	20.54	58.7	12.75	[100]
Cu_2_O	CH_3_NH_3_PbI_3−_ *_x_* Cl*_x_*	FTO/TiO_2_/CH_3_NH_3_PbI_3−_ *_x_* Cl*_x_*/Cu_2_O/Au	Magnetron Sputtering	0.96	15.8	59	8.93	[69]
Cu_2‐x_GeS_3_	CH_3_NH_3_PbI_3_	FTO/TiO2/ CH_3_NH_3_PbI_3_/ Cu_2‐x_GeS_3_/Au	Spin‐coating	1.03	18.55	63.80	12.56	[83]
CuGaO_2_	CH_3_NH_3_PbI_3−_ *_x_* Cl*_x_*	FTO/c‐TiO2/CH_3_NH_3_PbI_3−_ *_x_* Cl*_x_*/CuGaO_2_/Au	Spin‐coating	1.11	21.66	77	18.51	[70]
Cu_2_O	CH_3_NH_3_PbI_3_	FTO/ZnO/ CH_3_NH_3_PbI_3_/Cu_2_O/Au	Spin‐coating	0.76	12.58	63	6.02	[101]

#### 3.1.1 Copper (I) Oxide (Cu_2_O)

Copper (I) oxide (Cu_2_O) is a p‐type semiconductor with a direct bandgap energy of about 2.1 eV, which was first discovered as a potential candidate for photovoltaics in 1978 [63, 64]. It has low electron affinity and high hole mobility of around 100 cm^2^/(V.s) [65, 66]. Solution‐processed Cu_2_O nanocubes has been reported in n‐i‐p device configuration as HTL with a PCE of 17.23%. The device retains 96% of its initial efficiency after 500 h and 86% after 1000 h of continuous illumination under a relative humidity of 55% [67]. Cu_2_O quantum dots (QD) prepared in aqueous solution with surface modification using silane coupling agent (ethenyltriethyloxysilane) has been reported to induce hydrophobic properties with a water contact angle of 88.2°.

**FIGURE 4:**
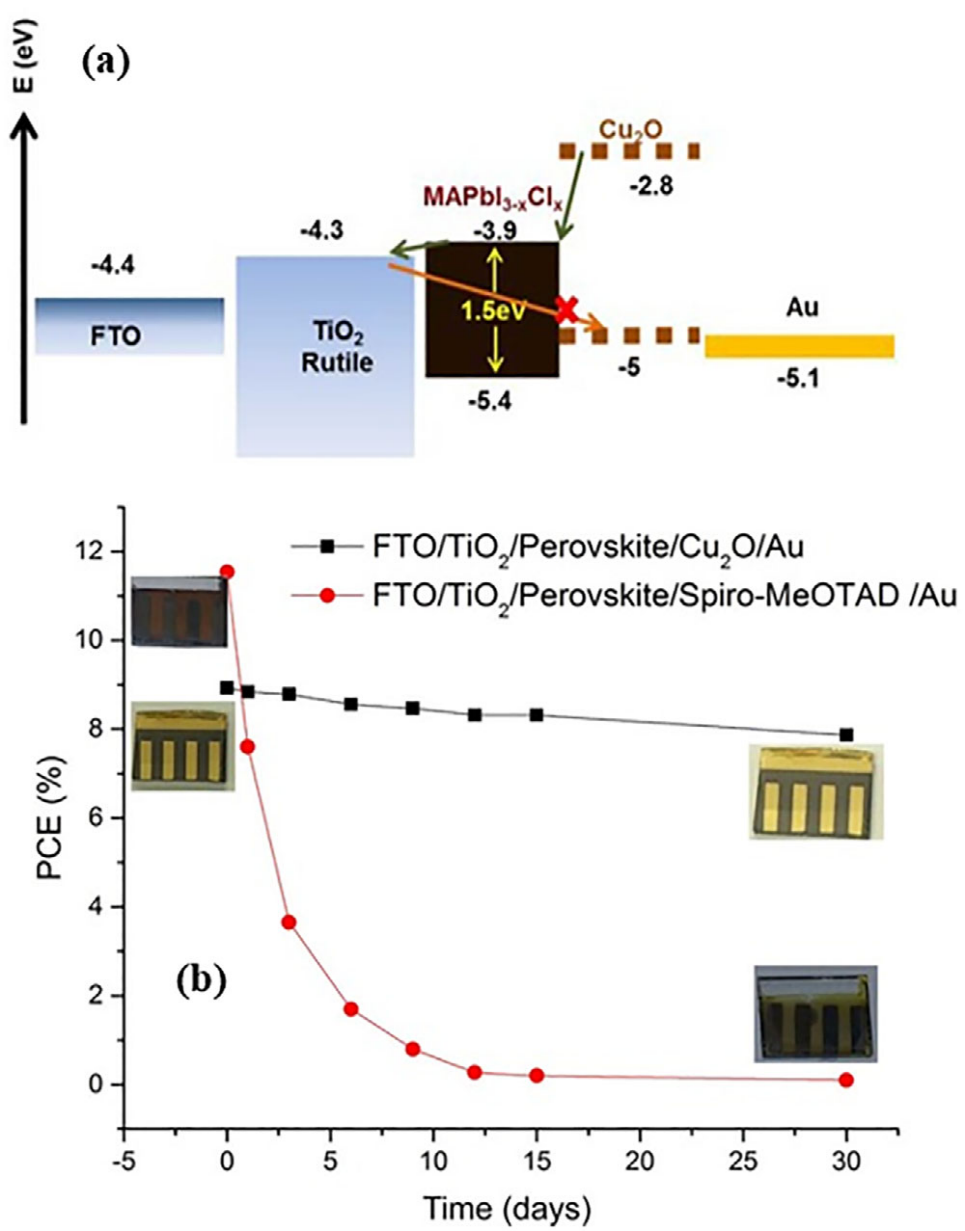
PSCs based on organic (Spiro‐OMeTAD) and inorganic (Cu_2_O) HTLs: (a) their energy‐level, and (b) device performance and stability with time. Reproduced with permission [69]. 2025,Wiley.

The surface modification of the Cu_2_O QD results in the improvement of PSC efficiency of 18.9%, and over 90% of the initial efficiency was maintained after 30 days of exposure of the device in ambient atmosphere [68]. The excellent device stability indicates that the hydrophobic properties of the Cu_2_O can efficiently prevent water penetration into the perovskite layer [68]. To further improve the stability of PSC using Cu_2_O HTL, Nejand et al. [69] directly deposited Cu_2_O on top of pin‐hole free perovskite layer by sputtering. Compared with a PSC having Spiro‐OMeTAD as HTL (Figure 4a), the device prepared using Cu_2_O shows a relatively lower PCE of 8.93% whereas 11.5% was obtained using Spiro‐OMeTAD. However, the device was extremely stable with negligible power loss even after 30 days while the Spiro‐OMeTAD based device degraded to 0% in 12 days as shown in Figure 4b [69].

Other than that, to create stable and efficient PSCs, solution‐processed copper‐based ternary metal oxides HTL including CuGaO_2_, CuCrO_2_ and CuFeO_2_ [70, 71, 72] have been reported. CuGaO_2_ are considered beneficial for semi‐transparent PSCs mainly because of improved transparency with better hole movement and low VBM [73, 74]. This transparent HTL appeared to promote the charge carrier and device stability and thereby reduce the parasitic heating in PSCs. According to Zhang et al., CuGaO_2_ device showed better hole selectivity due to its smaller ideality factor of 1.22 as compared to 1.48 of Spiro‐OMeTAD. The authors reported an average PSC efficiency of 15.9% and 17.2% for the spiro‐OMeTAD and CuGaO_2_, respectively [70].

#### 3.1.2 Copper Sulphide (CuS)

Copper sulphide (CuS) is a semiconductor with a direct bandgap energy ranging between 1.55 and 2.15 eV. This material has several applications in the field of chemical sensing, photovoltaics and thermoelectric materials [75, 76]. It has been reported as a low‐cost and stable HTL alternative to the conventional n‐i‐p PSCs. Tirado et al. reported spin‐coated CuS nanoparticles dispersed in polar solution as HTL with a remarkable PCE of 13.47% with MAPbI_3_ as the perovskite layer. The device shows a high value of J_sc_ and FF but a significant reduction in V_oc_ which is attributed to the recombination effect at the CuS/perovskite interface [77].The PSCs using MAPbI_3_ and CsFAMAPbIBr as light‐absorbing layers and CuS nano‐particles as HTL showed efficiency of 13.45% and 11.85%, respectively, with improved stability.

**FIGURE 5:**
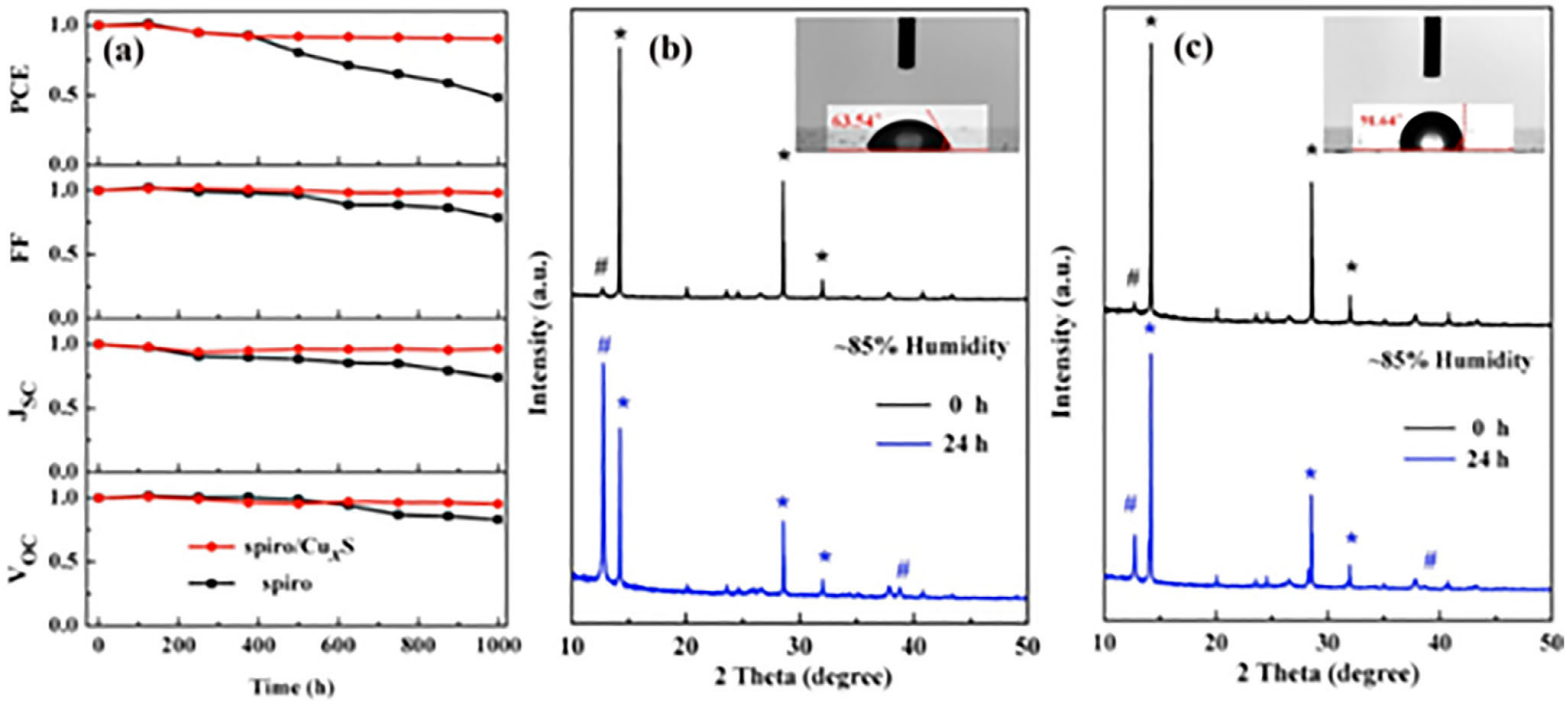
(a) PCE, FF, Jsc, and Voc as a function of ambient storage time of devices with spiro and spiro/Cu_1.75_S HTLs. The crystallinity of perovskite with (b) the spiro HTL, and (c) the spiro/Cu_1.75_S HTL. Reproduced with permission [79] 2025,Wiley.

Incorporating different stoichiometry of copper sulphide as an interlayer with organic HTL has also been reported to improve the overall PCE as well as the stability of the n‐i‐p PSC. Adding a layer of Cu_9_S_5_ nanoparticles with 12 nm thickness on Spiro‐OMeTAD (thickness ∼ 128 nm) results in a reduction of *J*–*V* hysteresis curve. This improves the stability of the cell, which maintained 96% of the initial efficiency (17.10%) after 1200 h [78]. Similarly, Cu_1.75_S with Spiro‐OMeTAD has been reported by Lei et al. [79] as a stable and efficient hole transport material. The deposition of 20 nm interlayer Cu_1.75_S above Spiro‐OMeTAD using thermal evaporation provides a protective layer from moisture (water contact angle of 91.6°), which results in retaining 90% of cell efficiency after 1000 h of storing the device in air, as shown in Figure 5. The overall device efficiency and V_oc_ also increased from 17.34% to 18.58% after adding the Cu_1.75_S layer on Spiro‐OMeTAD [79].

Like ternary copper oxides, ternary copper sulphide‐based materials for hole transport layer have been considered as an emerging material with a wide stability range and excellent optical absorption co‐efficient [80, 81]. With chemically fabricated copper antimony sulfide (Cu_12_Sb_4_S_13_), the reported power conversion efficiency was 6.5%. However, the device was able to retain its 50% efficiency for 15 days owing to the hydrophobicity of the HTM [82]. Similarly, using Cu_2‐x_GeS_3,_ the efficiency of the solar cell device has been enhanced to 12.56% and maintained 95% power conversion efficiency. However, the device using spiro‐OMeTAD as the hole‐transporting material experiences significant degradation within 2 days and almost complete degradation within 10 days [83].

Heidariramsheh et al., [84] reported the use of Cu_2_SnS_3_ nano‐particles as HTL. The materials were deposited using spin‐coating in various forms, including zincblende and wurtzite based on the use of thiols during the synthesis process. The article reported that the transformation of the crystalline structure of Cu_2_SnS_3_ NPs from zincblende to wurtzite causes a shift in the valence band energy, leading to an increase in the bandgap energy. This incompatible energy level of the zincblende Cu_2_SnS_3_ NPs HTM with the metal contact and the perovskite resulting in poor hole transport. The reference solar cell using spiro‐OMeTAD exhibited an efficiency of 16.01%, while the solar cell made with wurtzite‐ Copper Tin Sulphide (CTS) demonstrated a promising efficiency of 13.1% PCE, compared to 7.87% for the zincblende‐CTS. Impedance spectroscopy shows more charge recombination at the perovskite/HTM junction for wurtzite CTS as compare to spiro‐OMeTAD, which can be a key feature for further study to attain improved efficiencies [84].

#### 3.1.3 Copper Iodide (CuI)

**FIGURE 6:**
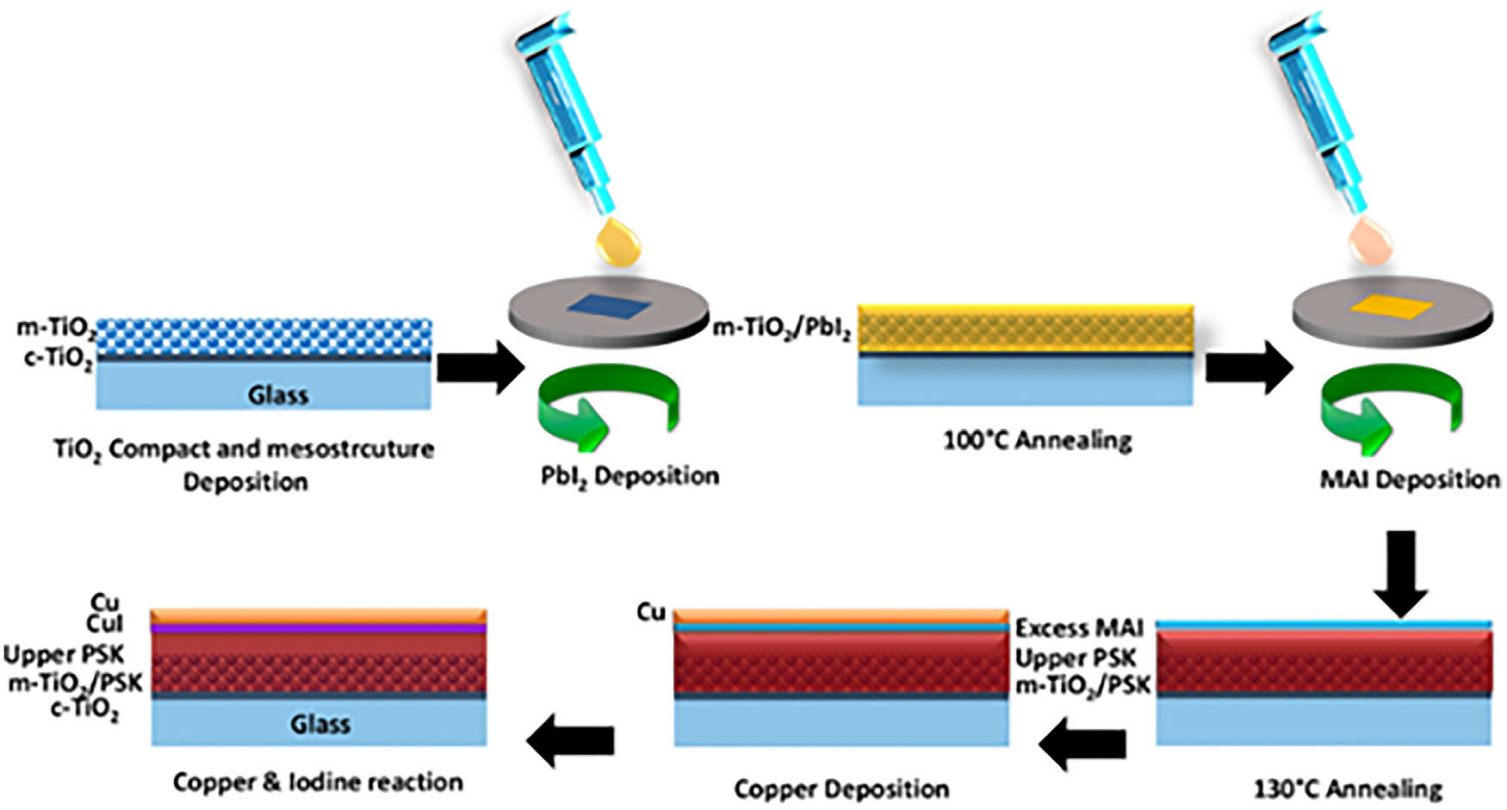
Fabrication steps for n‐i‐p PSCs with CH_3_NH_3_PbI_3_ as absorbing layer, including interface engineering of HTL using CuI Reproduced with permission [89]. Copyright 2025, ACS Publications.

Copper iodide (CuI) is a highly transparent p‐type semiconductor with a wide bandgap energy of ≈3.1 eV. It provides excellent valence band position and compatibility with solution processing perovskites [85, 86, 87]. CuI as HTM in *n‐i‐p* perovskite solar cell has been reported with a power conversion efficiency of 6.0% and improved *J*_sc_ and *FF* due to the high conductivity of the material. However, in comparison to spiro‐OMeTAD, it shows lower *V*_oc_ which was attributed to the high recombination rate across the perovskite/HTM interface [88]. Nazari et al. [89], reported interface engineering of HTL using CuI/Cu (FTO/TiO_2_/CH_3_NH_3_PbI_3_/CuI/Cu) to reduce the hole‐electron recombination at the perovskite/HTM interface. The CuI/Cu was created by annealing the thermally deposited Cu on the perovskite at 100°C for 10 min as shown in Figure 6. The PCE of the device was improved to 9.24% with hysteresis‐free photovoltaic properties alongside long‐term durability [89]. Reduced graphene oxide (rGO) has been applied as an interlayer due to its higher electrical and thermal conductivity to reduce the interfacial recombination and enhance hole extraction. Shi et‐al [90] reported interfacial engineering of HTL (CuI) using rGO with the device structure of FTO/TiO_2_/perovskite/rGO/CuI/rGO/Au. The prepared solar cell with optimal HTL (rGO/CuI/rGO) thickness of 200 nm resulted in improved *J*_sc_ from 15.63 to 18.86 mAcm^−2^ and PCE from 8.07% to 8.69% as compared to the PSC without the rGO interlayer [90]. Srivastave et al., [91] reported a unique method of using transfer‐printed CuI to reduce the deterioration of the surface of perovskite due to solution‐processed CuI. A rapid decline in the PCE from 8.3% to 6.6% is observed when the transfer printing temperature (T_TP_) increases above 80°C, mainly due to the degradation of the perovskite/CuI interface or the perovskite itself [91].

Table 1 summarises the PCE of PSCs using various oxides, sulphides and iodides of copper as the hole transport layer and their deposition methods. The table shows the photovoltaic performance of copper‐based HTL which with significant variation of V_oc_ (0.42–1.13 eV) and FF (31%–77%). The variation is primarily based on interfacial energy levels, film morphology, deposition method and defect density across the perovskite/HTL interface. For instance,

1. Copper oxide‐based HTL generally demonstrate high Voc (> 1.0 eV) and stable fill factor due to better energy alignment and reduced recombination pathways [67].
2. CuS‐based HTL exhibits lower Voc but reasonable FF (in the range of 70%), which is linked to interfacial recombination losses [77].
3. CuI‐based devices show a widespread value of V_oc_ (0.42–0.85 V) and in FF (31%–62%), reflecting strong dependence on deposition methods and interface quality, including degradation induced during solution processing or thermal treatment [87, 90].

However, most of the HTLs have been deposited using chemical solution methods, and there is a research gap using physical methods, including but not limited to sputter deposition, thermal and electron beam evaporation, as well as laser ablation.

### 3.2 Nickel Based HTL

**FIGURE 7:**
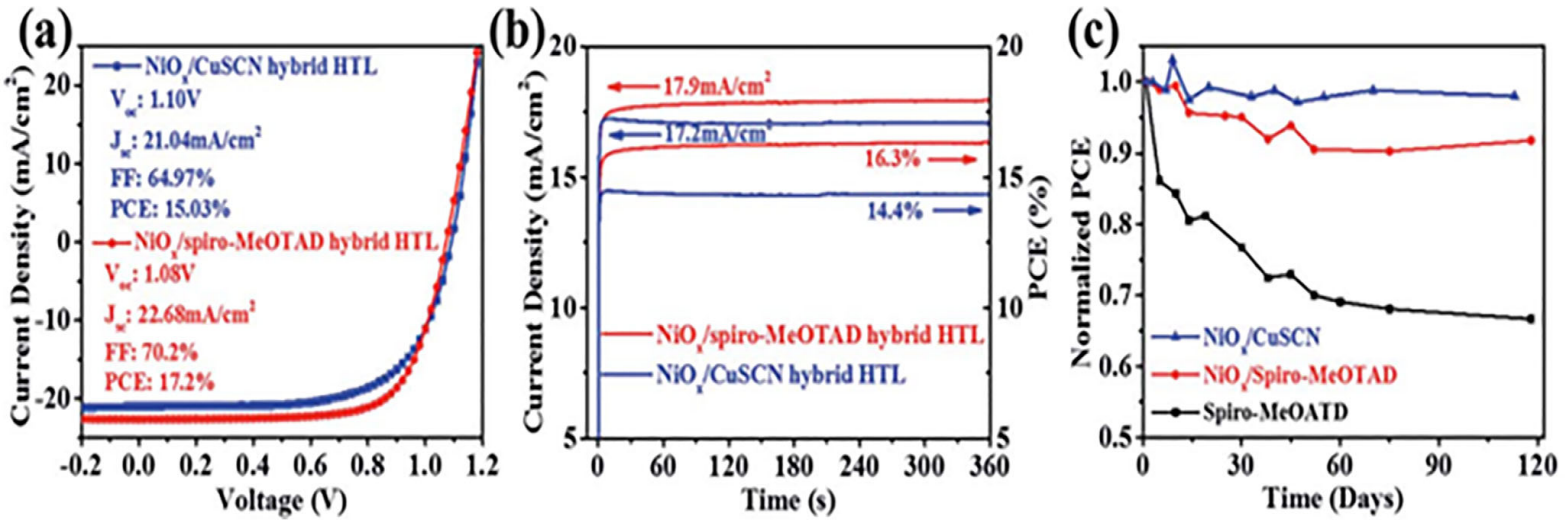
NiO_x_/CuSCN and NiO_x_/spiro‐MeOTAD hybrid HTLs for the best performing PSC devices. (a) Current density‐voltage (*J*–*V*) curves, (b) steady‐state current density and PCE at bias voltage of 0.84 V (NiO/CuSCN) and 0.91 V (NiOx/spiro‐MeOTAD), and (c) PCE of the device under humidity level of 50%–60% Reproduced with permission [113]. Copyright 2025, RSC Publications.

Nickel oxide is a p‐type semiconductor with a wide direct bandgap of 3.6–4.0 eV. It has been used in PSCs due to its excellent energy level alignment with most of the perovskite materials, suitable valence band maximum, chemical stability, superior electron blocking capability, and capacity to prevent reaction with perovskite [102, 103, 104, 105]. As shown in Table 2, NiO_x_ and its derivatives have been reported as substitutes for organic HTLs such as Spiro‐OMeTAD in n‐i‐p PSCs. NiO_x_ has been prepared using different methods in different structural forms, including NiO_x_ quantum dots, mesoporous NiO_x_, NiO_x_ nanoparticles and NiO_x_ nanocrystals [106, 107, 108, 109, 110, 111, 112]. Spin‐coating has been widely used, as reported by Cao et al. [113]. The device shows excellent chemical stability with the perovskite absorber in air for 4 months without encapsulation. The overall PCE of 5 wt.% NiO_x_ NP suspension was reported as 9.351%. This lower value was due to leakage current and defect states by nickel oxide, resulting in recombination. This issue was addressed by creating an innovative layer‐by‐layer structure based‐on an inorganic‐inorganic hybrid and inorganic‐organic hybrid HTL. Figure 7 shows the performance of PSCs using NiO_x_/CuSCN and NiO_x_/spiro‐MeOTAD hybrid HTLs with improved air stability (no‐degradation for 4 months without capsulation), higher efficiency (17.2%) and improved conductivity by providing better charge transport [113]. However, explicate report between encapsulated and non‐encapsulated devices is critical for long‐term use of the PSC devices.

**TABLE 2:** Nickel‐based HTL in PSCs.

Hole transport layer	Photoactive layer	Cell structure	Deposition method	V_oc_ (V)	J_sc_ (mA/cm^2^)	FF (%)	PCE (%)	Refs.
Hydrophobic nickel oxide nanocrystals	CsFAMAPbBrI	ITO/SnO_2_/CsFAMAPbBrI/NiO_x_/Al	Spin‐coating	1.01	22.35	50.41	11.38	[110]
NiO doped perovskite	NiO‐CH_3_NH_3_PbI_3_	ITO/M‐TiO_2_/ NiO‐CH_3_NH_3_PbI_3_/C	Hydrothermally prepared NiO, spin coated with perovskite	0.95	22.79	64	13.43	[126]
NiO@CSs Composite	CH_3_NH_3_PbI_3_	ITO/c‐TiO2/ CH_3_NH_3_PbI_3_/NiO@CSs	Doctor‐blade	0.84	22.054	63.11	11.70	[114]
P3HT/m‐NiOx	FA_0.6_MA_0.4_PbI_3_	ITO/SnO_2_/FA_0.6_MA_0.4_PbI_3_/P3HT/m‐NiO_x_/C	Spin‐coating	1.17	24	70	20.14	[112]
NiO QDs	CH_3_NH_3_PbI_3_	FTO/c‐TiO2/ CH_3_NH_3_PbI_3_/NiO/Au	Spin‐coating	1.02	10.77	56	6.2	[106]
NiOx‐HA	CH_3_NH_3_PbI_3_	FTO/TiO_2_/CH_3_NH_3_PbI_3_/NiO_x_‐HA/Au	Spin‐coating	0.99	21.9	60	13.1	[109]
NiO nanoparticles	CH_3_NH_3_I	FTO/m‐TiO_2_/CH_3_NH_3_I/NiO NP	Spin‐coating	0.97	18.12	62	10.89	[108]
NiO	CH_3_NH_3_PbI_3_	FTO/ZnO/ CH_3_NH_3_PbI_3_/Ni/Au	Spin‐coating	0.69	10.86	67	5.02	[101]
NiCo_2_O_4_	Cs_0.10_(FA_0.4_MA_0.6_)_0.9_PbI_2.8_Br_0.2_	FTO/c‐TiO_2_/m‐TiO_2_/Cs_0.10_(FA_0.4_MA_0.6_)_0.9_PbI_2.8_Br_0.2_/ NiCo_2_O_4_/Au	Spin‐coating	1.01	14.03	69.55	14.03	[119]
Sputtered NiO	(FA_0.83_MA_0.17_)_0.95_Cs_0.05_PbI_2.5_Br_0.5_	FTO/SnO_2_/(FA_0.83_MA_0.17_)_0.95_Cs_0.05_PbI_2.5_Br_0.5_/Sputtered NiO/Au	Sputtering	0.668	17.36	38.9	4.51	[122]
NiO_x_	FAMAPb(IBr)_3_	FTO/ZnTiO_3_/FAMAPb(IBr)_3_/NiOx/Au	Spin‐coating	1.083	23.62	73.32	18.75	[118]
NiO_x_ QD	(Cs_0.03_FA_0.97_PbI_3_)_0.95_(MAPbBr_3_)_0.05_	FTO/SnO_2_/(Cs_0.03_FA_0.97_PbI_3_)_0.95_(MAPbBr_3_)_0.05_/NiOx/Au	Spin‐coating	0.783	23.04	58.1	10.34	[120]
Mg:NiO_x_	(Cs_0.03_FA_0.97_PbI_3_)_0.95_(MAPbBr_3_)_0.05_	FTO/SnO_2_/(Cs_0.03_FA_0.97_PbI_3_)_0.95_(MAPbBr_3_)_0.05_/Mg:NiOx/Au	Spin‐coating	0.806	24.21	62.8	12.41	[120]
f‐NiO_x_ + CNT	FA_0.83_Cs_0.17_PbI_2.5_Br_0.5_	FTO/TiO_2_/FA_0.83_Cs_0.17_PbI_2.5_Br_0.5_/ f‐NiOx + CNT/C	Spin‐coating	0.97	18.30	64	11.36	[125]
QDs+NiO_x_	FAMAPb(IBr)_3_	FTO/ZnTiO_3_/FAMAPb(IBr)_3_/ CsPbIxBr1−x QDs/NiOx/Au	Spin‐coating	1.114	24.45	79.29	21.59	[118]
NiO_x_	(Cs_0.03_FA_0.97_PbI_3_)_0.95_(MAPbBr_3_)_0.05_	FTO/SnO_2_/(Cs_0.03_FA_0.97_PbI_3_)_0.95_(MAPbBr_3_)_0.05_/NiOx/Au	Spin‐coating	0.972	22.73	57.2	12.63	[127]
Li:NiO_x_	(Cs_0.03_FA_0.97_PbI_3_)_0.95_(MAPbBr_3_)_0.05_	FTO/SnO_2_/(Cs_0.03_FA_0.97_PbI_3_)_0.95_(MAPbBr_3_)_0.05_/Li:NiOx/Au	Spin‐coating	0.995	23.59	58.9	13.84	[127]
Mg‐Li:NiO_x_	(Cs_0.03_FA_0.97_PbI_3_)_0.95_(MAPbBr_3_)_0.05_	FTO/SnO_2_/(Cs_0.03_FA_0.97_PbI_3_)_0.95_(MAPbBr_3_)_0.05_/Mg‐Li:NiOx/Au	Spin‐coating	1.038	24.31	64.1	16.20	[127]
NiO_x_‐UVO	CH_3_NH_3_PbI_3_	FTO/c‐TiO_2_/m‐TiO_2_/ CH_3_NH_3_PbI_3_/NiO_x_/Au	Spin‐coating	0.881	19.49	53.1	9.11	[107]
Oil‐NiO_x_	(Cs_0.03_FA_0.97_PbI_3_)_0.95_(MAPbBr_3_)_0.05_	ITO/SnO_2_/(Cs_0.03_FA_0.97_PbI_3_)_0.95_(MAPbBr_3_)_0.05_/Oil‐NiO_x_/Ag	Meniscus blade printing	1.18	25.50	80.01	24.06	[128]
Oxygen plasma/NiO	CH_3_NH_3_PbI_3_	FTO/dense TiO_2_/m‐TiO_2_/ CH_3_NH_3_PbI_3_/NiO/Ag	Spin‐coating	0.93	16.72	39.3	6.10	[105]
NiCl_2_‐NiO_x_ NP	CH_3_NH_3_PbI_3_	FTO/b‐TiO_2_/m‐TiO2/CH_3_NH_3_PbI_3_/NiCl_2_‐NiO_x_/Au	Spin‐coating	0.885	23.59	60.10	12.57	[111]
PEG‐NiO_x_ NP	CH_3_NH_3_PbI_3_	FTO/b‐TiO_2_/m‐TiO2/CH_3_NH_3_PbI_3_/PEG‐NiOx/Au	Spin‐coating	0.834	20.71	57.10	9.87	[111]
PVP‐NiO_x_ NP	CH_3_NH_3_PbI_3_	FTO/b‐TiO_2_/m‐TiO2/CH_3_NH_3_PbI_3_/PVP‐NiOx/Au	Spin‐coating	0.859	12.10	57.30	10.55	[111]
Reflux‐NiO_x_ NP	CH_3_NH_3_PbI_3_	FTO/b‐TiO_2_/m‐TiO2/CH_3_NH_3_PbI_3_/Reflux‐NiOx/Au	Spin‐coating	0.834	21.68	53.20	9.62	[111]
Spiro‐OMeTAD+C@NiO	CsFAMA	ITO/SnO_2_/CsFAMA/C@NiOx/Spiro‐OMeTAD/Au	Spin‐coating	1.17	24.98	77	22.50	[129]
NiO_x_/CuSCN	MAFAPbBrI_3_	FTO/TiO_2_/ MAFAPbBrI_3_/ NiO_x_/Au	Spin‐coating	1.10	21.04	64.97	15.03	[113]
NiOx/Spiro‐OMeTAD	MAFAPbBrI_3_	FTO/TiO_2_/ MAFAPbBrI_3_/ NiOx/Spiro‐MeOTAD/Au	Spin‐coating	1.08	22.68	70.2	17.2	[113]
Tailored NiO NC	CsPbI_2_Br	FTO/Nb‐doped TiO_2_/CsPbI_2_Br/tailored NiO NC/Au	Spin‐coating	1.234	15.04	76.7	14.25	[130]
NiOx/Spiro	CsPbI_2_Br	FTO/c‐TiO_2_/m‐TiO_2_/ CsPbI_2_Br/NiOx/Spiro/Au	Spin‐coating	1.25	14.26	76	13.6	[131]
NiOx/Sprio	PVSK	ITO/SnO_2_/PVSK/NiO_x_/Spiro/Au	Spin‐Coating	1.14	23.82	79.8	21.66	[117]
NiO/Spiro‐OMeTAD	CH_3_NH_3_PbI_3_	FTO/TiO_2_/ CH_3_NH_3_PbI_3_/NiO/Spiro‐OMeTAD/Ag	Spray Method/Spin‐coating	0.97	21.1	53.45	10.95	[124]
NiO@C/Spiro‐OMeTAD	PVSK	FTO/c‐TiO_2_/mp‐TiO2/PVSK/NiO@C/Spiro‐OMeTAD/Au	Spin‐Coating	1.018	22.39	69.24	15.78	[116]
NiO	CH_3_NH_3_PbI_3_	FTO/c‐TiO2/mp‐TiO_2_/ CH_3_NH_3_PbI_3_/NiO/C	Screen‐printing	0.89	18.2	71	11.4	[115]

Sajid et al. [114] reported the use of NiO alongside carbon to make a composite for the contact to prepare a low‐cost perovskite solar cell by removing Au as the top electrode. This composite of NiO@carbon sphere not only improves the energy level between the perovskite and the metal contact, but also provides a better electron blocking layer, leading to enhanced *J*_sc_ and *FF* with an overall PSC of 11.70% [114]. Similarly, adding a small layer of screen‐printed NiO between the electrode and the perovskite is reported to improve by providing better hole movement and electron blocking, contributing to an improvement in efficiency of 11.4% [115]. Adding a combination of NiO and C in between the perovskite and Spiro HTL is reported to reduce the charge transport and charge recombination resistance and improve the photovoltaic parameters and PCE of 15.78% [116].

Li et al. reported the NiOx/Spiro layer as HTL on perovskite with excellent efficiency of 21.66% by improving the hole extraction due to improved energy level between the interface with perovskite (E_VB_ of NiOx as 5.27 eV and HOMO energy level of Spiro is around 5.2 eV) and retaining the efficiency to 90% for the first 30 h (4 times better than Spiro‐OMeTAD only based HTL) provide a better comparison between stability improvement based on inorganic HTL [117]. Similarly, adding an interface of spin‐coated NiOx QD between the perovskite and NiO_x_ HTL exhibits high efficiency of 21.59% with enhanced stability during 85°C aging testing and is considered as an excellent HTL for semi‐transparent perovskite solar cells [118]. It should be noted that variations in testing protocols and reporting standards make direct comparison of device stability across different studies challenging.

A mixed metal oxide of NiCo_2_O_4_ nanoparticles has been reported as HTL with a PCE value of more than 14% [119]. Further improvement in efficiency was observed when an interfacial layer of Spiro‐OMeTAD was deposited between NiCo_2_O_4_ layer and Au electrode. The advantage of using the mixed metal oxide as an interface layer includes a reduction in charge recombination and providing a pathway to reduce the grain boundaries created due to metal electrode deposition [119].

Most of the NiO_x_ based HTL in Table 2 were prepared by solution processing. Since solution processing of nickel oxide requires polar solvents, evaporation of the polar solution at high temperature (150–350°C) can affect the perovskite layer beneath it. To reduce this effect, Guo et al. [120] prepared the NiO_x_ nanocrystals soluble in polar solvent that don't require annealing of the HTL, resulting in improved efficiency of 10.34% and enhanced stability. With the addition of magnesium in NiO_x_, the authors further improved the efficiency of the PSC from 10.34% to12.41% by adjusting the energy level of the HTL [120]. As the fabrication process of the NiO_x_ as HTL is generally limited to spin‐coating and screen printing, development of the HTL using vacuum deposition techniques (e.g. e‐beam evaporation, sputtering, pulsed layer deposition) is an avenue for future research. Deposition of NiO_x_ HTL using plasma processing, including sputtering, is reported to affect the perovskite layer below the HTL [121]. Basak et al. demonstrated the use of RF sputtered NiO_x_ as the HTL for halide perovskite solar cell and reported that the defects were significantly healed (however, not completely) with time, resulting 180%–220% improvements of their initial PEC value in about 9 days [122]. Bi‐layer inorganic HTL, including NiO_x_/CuSCN and Cs:NiO_x_/CuSCN achieved excellent PCE of 16.58% and 18.42%, respectively. This bi‐layer also protecting the perovskite from polar diethyl sulfide solvent and improved both the fill factors and extraction of holes [123].

Table 2 complies the photovoltaic parameters of NiO_x_ based HTLs with a wide variation of Voc and FF, which is attributed to the film quality, defect density and interfacial energy alignment with the perovskite. The observation depicts that the performance of NiOx based HTL is highly sensitive to processing conditions, doping strategies and interface engineering. For instance, NiO sputtered over perovskite shows relatively lower FF (38.9%) associated with poor film uniformity and interface damage during deposition [122]. In contrast, higher FF and V_OC_ values observed in doped or composite systems (e.g., Mg:NiO_x_, NiO_x_/Spiro, or NiO_x_/CNT) can be attributed to improved energy level alignment, enhanced hole mobility, and reduced interfacial recombination [120, 124, 125]. Furthermore, surface passivation and interfacial engineering strategies, such as the incorporation of quantum dots or bilayer HTLs, have been shown to significantly improve charge extraction and suppress recombination losses.

Overall, while these studies indicate improved device stability with different NiO_x_ structure and bi‐layer structures, it should be noted that many such results are based on short‐term, non‐standardized testing conditions, and often do not clearly distinguish between encapsulated and unencapsulated devices, limiting direct comparability across reports.

### 3.3 CuSCN Based HTL

Copper (I) Thiocyanate (CuSCN) is a p‐type semiconductor with a wide bandgap energy (typically 3.6 eV) categorized as HTL in PSCs due to its high hole mobility and improved transparency [132, 133]. CuSCN based HTLs are mainly prepared using solution processing as shown in Table 3. Qin et al. reported doctor blading coated CuSCN, which yields a solar cell efficiency of 12.4% with improved short circuit current that opens the door for low processing‐ cost HTL in planar PSCs [134]. However, CuSCN can dissolve in propyl sulfide and partly redissolve the perovskite absorber, causing a short circuit between the ETL (TiO_2_) and HTL (CuSCN) [135]. Therefore, engineering the perovskite absorber or CuSCN layer is required to reduce the unfavorable charge recombination [134]. Mashhoun et al. reported Ta‐WO_x_ as an interlayer between CuSCN and carbon electrode, which was found to improve the PCE of the device from 8.59% to 12.31% [136].

**TABLE 3:** CuSCN based HTL in PSCs.

Hole transport layer	Photoactive layer	Cell structure	Deposition method	V_oc_ (V)	J_sc_ (mA/cm^2^)	FF (%)	PCE (%)	Refs.
EA treated CuSCN	CH_3_NH_3_PbI_3_	ITO/SnO_2_/ CH_3_NH_3_PbI_3_/EA treated CuSCN/Au	Spin‐coating	0.99	22.62	70.9	15.86	[137]
CuSCN	CH_3_NH_3_PbI_3_	ITO/SnO_2_/ CH_3_NH_3_PbI_3_/CuSCN/Au	Spin‐coating	0.97	22.13	68.7	14.72	[137]
CuSCN	CH_3_NH_3_PbI_3_	FTO/TiO_2_/CH_3_NH_3_PbI_3_/CuSCN/Au	Spin‐coating	0.83	22.15	60	11.02	[145]
CuSCN	MA_0.5_FA_0.5_PbI_3‐x_Cl_x_	FTO‐glass/compact TiO_2_ /MA_0.5_FA_0.5_PbI_3‐x_Cl_x_/CuSCN/Au	Spin‐coating	1.03	21.40	61.2	13.49	[138]
PDMS/CuSCN	MAPbI_3_	ITO/SnO_2_/MAPbI_3_/PDMS/CuSCN/Au	Spin‐coating	1.02	23.90	78.30	19.04	[139]
CuSCN/rGO	MAFAPbBrI_3_	FTO/TiO2/ MAFAPbBrI_3_/CuSCN/rGO/Au	Drop‐casting	1.11	23.24	78.20	20.40	[141]
CuSCN/DTB	PVSK	ITO/SnO2/PVSK/CuSCN/DTB/Au	Drop casting	1.15	24.31	78.58	22.0	[140]
CuSCN/Ta‐WOx	CH_3_NH_3_PbI_3_	ITO/SAM/ CH_3_NH_3_PbI_3_/CuSCN/Ta‐WO_x_/C	Screen printing	1.01	17.21	71	12.31	[136]
LiSCN doped CuSCN	CH_3_NH_3_PbI_3_	ITO/SnO_2_/ CH_3_NH_3_PbI_3_/LiSCN doped CuSCN/Au	Spin‐coating	1.03	22.62	69	16.13	[142]
CuSCN	CH_3_NH_3_PbI_3_	FTO/TiO_2_/CH_3_NH_3_PbI_3_/CuSCN/Au	Spin‐coating	0.93	17.2	63	10.1	[144]
CuSCN	CH_3_NH_3_PbI_3_	FTO/TiO_2_/CH_3_NH_3_PbI_3_/CuSCN/Au	Doctor Blade	1.02	19.7	62	12.4	[134]
CuSCN	MAFAPb(I,Br)_3_	FTO/TiO_2_/MAFAPb(I,Br)_3_/CuSCN/Au	Spin‐coating	1.04	23.1	75.3	18.0	[143]

To reduce the perovskite degradation due to CuSCN dissolved in propyl‐sulfide, several different modifications in the CuSCN deposition process have been reported. Anti‐solvent treatment of HTL with ethyl‐acetate results in better crystallinity of CuSCN and results in reduced degradation of perovskite with 15.86% reported efficiency [137]. Fan et al. described that the delay in annealing of the CuSCN by 10 mins could significantly improve the efficiency from 10.39% to 13.49%. The drying process before annealing results provide enough time for CuSCN to form a smooth film due to the slow evaporation of the solvent [138]. Similarly, interfacial engineering by incorporating organic HTL has been proposed to significantly improve the interface between perovskite and CuSCN [139, 140]. Adding an interlayer of reduced graphene oxide (rGO) between perovskite and CuSCN improved both the efficiency (20.2%) and stability (retaining around 85% of the initial PCE after 1000 h) [141]. Furthermore, LiSCN doping in CuSCN demonstrate improvement in the electrical properties and supress the damage of the diethyl sulfide solvent (in which CuSCN is dissolved) on the perovskite layer by interface engineering. An PCE as higher as 20.95% was obtained with LiSCN doping [142].

Table 3 shows variation in photovoltaic performance for CuSCN based HTL in n‐i‐p PSCs, which is mainly governed by processing strategies and interfacial engineering. The photovoltaic performance shows that pristine CuSCN shows significantly lower V_oc_ and FF due to solvent‐induced degradation losses and partial dissolution of the perovskite solar during HTL deposition [139, 143]. To reduce this issue, modified systems including CuSCN/rGO, CuSCN/DTB and PDMS/CuSCN exhibits improved FF (>78%) and results in suppressing the charge recombination pathway and improved interfacial passivation [144]. Similarly, LiSCN doping and anti‐solvent treatments improve crystallinity and electronic properties of CuSCN, leading to enhanced charge transport and device performance [142].

### 3.4 Multifunctional Hybrid Oxides

There are several other metal oxides, including chromium oxide, cobalt oxide, tungsten oxide, molybdenum oxide and vanadium oxide, which have been reported as multifunctional inorganic HTLs in planar (both regular and inverted) PSCs as summarized in Table 4. The metal oxides show poor conductivity and energy level alignment with the absorber layer. Doping engineering can help tailor the band alignment, resulting in less recombination around the absorber layer and HTL interface, which enhancing performance.

**TABLE 4:** Multi‐function‐based HTL in PSCs.

Hole transport layer	Photoactive layer	Cell structure	Deposition method	V_oc_ (V)	J_sc_ (mA/cm^2^)	FF (%)	PCE (%)	Refs.
Spiro‐OMeTAD/CrOx	FA_0.82_Cs_0.13_MA_0.05_Pb(I_0.80_Br_0.20_)_3_	ITO/SnO_2_/ FA_0.82_Cs_0.13_MA_0.05_Pb(I_0.80_Br_0.20_)_3_/Spiro‐OMeTAD/CrO_x_/ITO	Reactive thermal deposition	1.16	20.83	79.21	19.06	[147]
MWCNT/Cr_2_O_3_	CH_3_NH_3_PbI_3_	FTO/TiO_2_/ CH_3_NH_3_PbI_3_/MWCNT/Cr_2_O_3_/Au	Spin‐coating	1.003	20.80	78.1	16.29	[146]
CuCrO_2_/PTAA	Cs_0.05_(MA_0.15_FA_0.85_)_0.95_Pb(I_0.85_Br_0.15_)_3_	ITO/SnO_2_/Cs_0.05_(MA_0.15_FA_0.85_)_0.95_Pb(I_0.85_Br_0.15_)_3_/CuCrO_2_/PTAA/Au	Spin‐coating	1.02	22.8	75	17.4	[163]
CuCrO_2_‐Spiro‐OmeTAD	Cs_0.05_ (FA_0.85_MA_0.15_)_0.95_Pb(I_0.85_Br_0.15_)_3_	ITO/SnO_2_/Cs_0.05_ (FA_0.85_MA_0.15_)_0.95_Pb(I_0.85_Br_0.15_)_3_/CuCrO_2_‐Spiro‐OMeTAD/Ag	Spin‐coating	1.11	23.01	76	19.5	[164]
CrOx/Spiro‐OMeTAD	CH_3_NH_3_PbI_3_	ITO/SnO_2_/ CH_3_NH_3_PbI_3_/CrOx/Spiro‐OMeTAD/Ag	Spin‐coating	1.161	23.61	77.39	21.21	[161]
Co_3_O_4_	CH_3_NH_3_PbI_3_	FTO/TiO_2_/ZrO_2_/CH_3_NH_3_PbI_3_/Co_3_O_4_/C	Spin‐coating	0.95	23.11	53	11.68	[160]
WO_3_/Spiro‐OMeTAD	Cs_0.05_(FAPbI_3_)_0.79_(MAPbBr_2_)_0.16_	FTO/SnO_2_/Cs_0.05_(FAPbI_3_)_0.79_(MAPbBr_2_)_0.16_/WO_3_/Spiro‐OMeTAD/Ag	Spin‐coating	1.17	23.7	77.1	21.44	[148]
WO_3_	CsPbBr_3_	FTO/TiO_2_/ CsPbBr_3_/WO_3_/C	Spin‐coating	1.307	8.04	75	7.90	[165]
WO_2_	CsPbBr_3_	FTO/TiO_2_/ CsPbBr_3_/WO_2_/C	Spin‐coating	1.219	7.46	69	6.24	[165]
WO_2.72_	CsPbBr_3_	FTO/TiO_2_/ CsPbBr_3_/WO_2.72_/C	Spin‐coating	1.288	7.64	77	7.57	[165]
WO_3‐x_ nano‐rods /Spiro‐OMeTAD	CH_3_NH_3_PbI_3_	ITO/SnO_2_/ CH_3_NH_3_PbI_3_/ WO_3‐x_ nano‐rods/ Spiro‐OMeTAD/Ag	Spin‐coating	1.12	23.92	78.78	21.14	[162]
PEDOT: PSS+WO_3_	FA_0.4_MA_0.6_PbI_2.8_Br_0.2_	ITO/SnO_2_/FA_0.4_MA_0.6_PbI_2.8_Br_0.2_/PEDOT: PSS+WO_3_/MoO_3_/Ag	Spin‐coating	1.03	22.69	64.84	15.10	[166]
NiPc‐(OBu)_8_/V_2_O_5_	(FAPbI_3_)_0.85_(MAPbBr_3_)_0.15_	FTO/TiO_2_/(FAPbI_3_)_0.85_(MAPbBr_3_)_0.15_/NiPc‐(OBu)_8_/V_2_O_5_/Au	Spin‐coating	1.08	23.1	73.4	18.3	[153]
PTAA/VO_x_	PCBA/CsFAPbI_3_/MAI	ITO/SnO_2_/PCBA/CsFAPbI_3_/MAI/PTAA/VO_x_/Ag	Spin‐coating	1.04	24.6	78	20.1	[158]
Spiro‐TTB/VO_x_	Cs_0.05_MA_0.15_FA_0.8_Pb(I_0.85_Br_0.15_)_3_	ITO/a‐NbO_x_/ Cs_0.05_MA_0.15_FA_0.8_Pb(I_0.85_Br_0.15_)_3_/Spiro‐TTb/VO_x_/Ag	Spin‐coating	1.2	21.6	76.6	19.8	[167]
Spiro‐OMeTAD/V_2_O_5_	FA_0.95_Cs_0.05_Rb_0.01_PbI_3_	FTO/SnO_2_/FA_0.95_Cs_0.05_Rb_0.01_PbI_3_/Spiro‐OMeTAD/V_2_O_5_/Au	Spin‐coating	1.15	24.66	81.4	23.02	[154]
ZrO_x_/VO_x_	CH_3_NH_3_PbI_3_	FTO/TiO_2_/CH_3_NH_3_PbI_3_/ZrO_2_/VO_x_/C	Spin‐coating	0.71	22.3	70	15.56	[155]
PTAA/VO_x_	CH_3_NH_3_PbI_3_	ITO/SnO_2_:PCBA/ CH_3_NH_3_PbI_3_/PTAA/VO_x_/Al	Spin‐coating	1.03	22.2	77	16.1	[156]
VO_x_	CH_3_NH_3_PbI_3_	FTO/TiO_2_/ CH_3_NH_3_PbI_3_/VO_x_/C	Spin‐coating	0.98	18.26	63.23	11.28	[159]
Spiro‐TTB (Opaque)/VO_x_	Cs_0.17_FA_0.83_Pb(Br_0.17_I_0.83_)_3_	ITO/SnO_2_/ Cs_0.17_FA_0.83_Pb(Br_0.17_I_0.83_)_3_/Spiro‐TTB/VO_x_/Au	Thermal Evaporation	1.07	18.9	71	14.2	[168]
Spiro‐TTB (Semi‐transparent)/VO_x_	Cs_0.17_FA_0.83_Pb(Br_0.17_I_0.83_)_3_	ITO/SnO_2_/ Cs_0.17_FA_0.83_Pb(Br_0.17_I_0.83_)_3_/Spiro‐TTB/VO_x_/ITO	Thermal Evaporation	1.06	17.1	73	13.2	[168]
3EtCz/VO_x_	(FA_0.79_MA_0.16_Cs_0.05_)_0.97_Pb(I_0.84_Br_0.16_)_2.97_	ITO/SnO_2_/(FA_0.79_MA_0.16_Cs_0.05_)_0.97_Pb(I_0.84_Br_0.16_)_2.97_/3EtCz/VO_x_/IZO	Spin‐coating	0.96	19.28	64	11.98	[169]
PTAA/V_2_O_x_	Cs_0.08_FA_0.80_ MA_0.12_ Pb (I_0.88_ Br_0.12_)_3_	ITO/c‐TiO_2_/m‐TiO_2_/ Cs_0.08_FA_0.80_ MA_0.12_ Pb (I_0.88_ Br_0.12_)_3_/PTAA/V2Ox/Au	Spin‐coating	1.00	22.70	75.83	16.75	[157]
KFeO2	CH_3_NH_3_PbI_3_	FTO/ZnO/ CH_3_NH_3_PbI_3_/KFeO2/Pt	Spin‐coating	0.94	15.79	73	10.83	[170]
MoO2 & 5.0% PTAA	CsPbI_2_Br	FTO/c‐TiO_2_/CsPbI_2_Br/MoO_2_&PTAA/C	Spin‐coating	1.21	15.07	80.44	14.67	[171]
MoO2	CsPbI_2_Br	FTO/c‐TiO_2_/CsPbI_2_Br/MoO_2_/C	Spin‐coating	1.18	14.57	75.04	12.90	[171]
Fe_3_O_4_	CH_3_NH_3_PbI_3_	FTO/c‐TiO_2_/mp‐TiO_2_/ CH_3_NH_3_PbI_3_/Fe_3_O_4_/Au	Spin‐coating	1.13	19.3	71	15.4	[172]

Chromium oxide has been reported in the literature as a ternary metal oxide to improve the properties of HTL in favor of increasing hole mobility, tailoring energy band alignment and improving the thermal and chemical stability of the PSCs. By adding multi‐wall carbon nanotubes (MWCNTs) [137] in Cr_2_O_3_, the conductivity of the material has been improved, whereas the valance band energy level reduced from 5.54 to 5.32 eV. This shift makes the HTL valance band better aligned with the energy level of the perovskite, resulting in fast charge transfer and reduced recombination. Further, incorporating MWCNT in Cr_2_O_3_ boosted the hydrophobicity of the HTL, resulting in a stable device with PCE of 16.29% and suppressed hysteresis [146]. To reduce the degradation of Spiro‐OMeTAD during sputter deposition of ITO in a semi‐transparent PSCs, chromium oxide was used as a buffer layer between spiro‐OMETAD and the ITO [147].

Tungsten oxide (WO_3_) is a typical n‐type transparent semiconductor with a wide bandgap energy (2.6–3.2 eV). Incorporating of WO_3_ in Spiro‐MeOTAD films is reported to shift Spiro's HOMO binding energy toward the Fermi level of Cs_0.05_(FAPbI_3_)_0.79_(MAPbBr_2_)_0.16_ as perovskite, which benefits the hole‐hole transport between the perovskite and Spiro‐OMeTAD, substantially improved the *V*_OC _value from 1.09 V to 1.17 V and thus PCE up to 21.44% [148]. Molybdenum oxide (MoO_3_) is an n‐type semiconductor with a wide bandgap and high work function (>6 eV). Device simulation has shown that e‐beam evaporated MoO_x_ can be a promising hole transport material with an estimated efficiency of 18.25% [149]. Nanoparticles of MoO_3_ was introduced into organic HTL (PEDOT:PSS), achieving enhanced efficiency (19.64%) and greater stability with the PSCs [150].

Recently, vanadium oxide thin film has emerged as a promising HTL for PSCs. The material has multi‐oxidation states (V_2_O_3_, V_3_O_5_, VO_2_, V_3_O_7_, V_2_O_5_ as the most stable materials) and various crystalline structures that gives wider range of properties and applications. Vanadium oxide is known as an n‐type semiconductor, but depending on deposition conditions, it can be found as p‐type [151] and used as HTL for PSCs. V_2_O_5_ has been reported as HTL, as shown in Table 4. The material has a work‐function of 4.7 eV, bandgap energy of 2.4 eV, but lower conductivity and hole selectivity [152]. The lower conductivity and larger energy mismatch with the perovskite make this material challenging as an HTL. However, doping of V_2_O_5_ with some organic or inorganic HTL can be a way forward to improve the work function, hole selectivity, V_oc_, and bandgap tuning. Adding a thin layer of VO_x_ alongside the organic HTL is expected to suppress degradation of perovskite at elevated temperatures, improving stability by blocking moisture and oxygen and reduce damage caused by sputtering of the upper transparent electrode [153, 154, 155, 156, 157]. Marina et al. proposed solution‐processed PTAA/VO_x_ as a hole transport layer with better stability at higher temperatures and PCE more than 20% [158]. However, the thickness, deposition process and annealing temperature of the VO_x_ layer require close monitoring for efficient PSCs.

The energy alignment and conductivity limitation of the above‐listed multi‐functional hybrid materials contribution to the broad variation of V_oc_ and FF as shown in Table 4. Pristine oxides like VO_x_ and Co_3_O_4_ exhibits significantly lower FF and Voc due to poor intrinsic conductivity and suboptimal hole selectivity, which result in high recombination losses [159, 160]. In contrast, hybrid systems incorporating organic HTLs (e.g., Spiro‐OMeTAD/CrOx, WO_3_/Spiro‐OMeTAD, and Spiro‐OMeTAD/V_2_O_5_) demonstrate significantly improved FF (>75%) and higher V_OC_, which is attributed to enhanced interfacial energy levels, improved charge extraction, and reduced recombination pathways [158, 161, 162]. Furthermore, doping and composite strategies, such as the incorporation of MWCNTs in Cr_2_O_3_ or WO_3_ nanostructures, improve conductivity and band alignment, thereby enhancing device performance [146].

### 3.5 Chalcogenide, Sulphide and Organic‐Inorganic Based Materials

For carbon‐based PSCs, the functionalized reduced graphene oxide (rGO) as HTL demonstrate commendable PCE of 17% owing to reduction in charge recombination and reduced series resistance, exhibiting better charge transport and hole movement as shown in Table 5 [173]. However, the use of nano‐graphene and graphene oxide shows lower efficiencies (12.81% and 10.01%, respectively) mainly due to low fill factors and poor interfacial contact with the perovskite and low solubility in chlorobenzene [174, 175]. Chalcogenide‐based HTL including Cu (In,Ga)S_2_ and CuInS_2_ exhibits PCEs ranging from 10.85% to 15.58% depending on the gallium content. The increase in the gallium content changes the bandgap and provide a way to engineering the energy levels, achieving enhanced hole collection and reduced electron‐hole recombination [176].  Notably, blending CuIn_0.75_Ga_0.25_S_2_ with 10% Spiro‐OMeTAD boosted the PCE of PSCs to 18.72%, showing the advantage of interfacial engineering [177]. Solution processable metal sulphides have shown promising results as hole transport layer with Sb_4_S_3_ and Cu_3_SbS_4_ reaching over 14% efficiency, indicating the potential in scalable and stable device design [178].

**TABLE 5:** Chalcogenide, Metal sulphides and inorganic and organic composite‐based HTL for PSCs.

Hole transport layer	Photoactive layer	Cell structure	Deposition method	V_oc_ (V)	J_sc_ (mA/cm^2^)	FF (%)	PCE (%)	Refs.
AF‐rGO	CH_3_NH_3_PbI_3_	ITO/SnO_2_ /CH_3_NH_3_PbI_3_/AF‐rGO/Ag	Spin‐coating	1.04	23.3	70.3	17	[173]
CZTS‐MWCNT	CH_3_NH_3_PbI_3_	FTO/c‐TiO_2_/m‐TiO_2_/ CH_3_NH_3_PbI_3_/CZTS‐MWCNT/MWCNT	Spin‐coating	0.95	13.39	60	7.60	[182]
GO	CH_3_NH_3_PbI_3_	FTO/c‐TiO_2_/m‐TiO_2_/CH_3_NH_3_PbI_3_/GO/carbon	Spin‐coating	0.68	28.50	25.8	10.01	[174]
Nanographene	CH_3_NH_3_PbI_3_	FTO/TiO_2_/CH_3_NH_3_PbI_3_/nanographene/Au	Spin‐coating	0.95	20.56	65.79	12.81	[175]
Cu(I _0.75_Ga_0.25_)S_2_	Cs_0.05_ (MA_0.17‐_FA _0.83_)_0.95_ Pb(I_0.83_ Br_0.17_)_3_	FTO/c‐TiO_2_/m‐TiO_2_/ Cs_0.05_ (MA_0.17‐_FA _0.83_)_0.95_ Pb(I_0.83_ Br_0.17_)_3_/ Cu(I _0.75_Ga_0.25_)S_2_/Au	Spin‐coating	0.98	19.33	60	11.45	[176]
Cu(In_0.5_Ga_0.5_)S_2_	Cs_0.05_ (MA_0.17‐_FA _0.83_)_0.95_ Pb(I_0.83_ Br_0.17_)_3_	FTO/c‐TiO_2_/m‐TiO_2_/ Cs_0.05_ (MA_0.17‐_FA _0.83_)_0.95_ Pb(I_0.83_ Br_0.17_)_3_/ Cu(I _0.5_Ga_0.5_)S_2_/Au	Spin‐coating	1.05	21.67	69	15.58	[176]
Cu(In_0.25_Ga_0.75_)S_2_	Cs_0.05_ (MA_0.17‐_FA _0.83_)_0.95_ Pb(I_0.83_ Br_0.17_)_3_	FTO/c‐TiO_2_/m‐TiO_2_/ Cs_0.05_ (MA_0.17‐_FA _0.83_)_0.95_ Pb(I_0.83_ Br_0.17_)_3_/ Cu(I _0.75_Ga_0.25_)S_2_/Au	Spin‐coating	0.99	19.5	65	12.58	[176]
CuInS_2_	Cs_0.05_ (MA_0.17‐_FA _0.83_)_0.95_ Pb(I_0.83_ Br_0.17_)_3_	FTO/c‐TiO_2_/m‐TiO_2_/ Cs_0.05_ (MA_0.17‐_FA _0.83_)_0.95_ Pb(I_0.83_ Br_0.17_)_3_/ Cu(I _0.75_Ga_0.25_)S_2_/Au	Spin‐coating	0.97	18.44	60	10.85	[176]
CuNiTS	CH_3_NH_3_PbI_3_	FTO/m‐TiO_2_/CH_3_NH_3_PbI_3_/CuNiTS/Au	Spin‐coating	0.92	17.75	54	8.85	[183]
CuCoTS	CH_3_NH_3_PbI_3_	FTO/m‐TiO_2_/CH_3_NH_3_PbI_3_/CuCoTS/Au	Spin‐coating	0.87	16.87	50	7.31	[183]
CISe	PVSK	ITO/SnO_2_/PVSK/CISe/Au	Spin‐coating	0.979	20.46	68.5	13.72	[184]
CuIn_0.75_Ga_0.25_S_2_	Cs_0.05_FA_0.81_MA_0.14._PbI_2.55_Br_0.45_	FTO/c‐TiO_2_/m‐TiO_2_/Cs_0.05_FA_0.81_MA_0.14._PbI_2.55_Br_0.45_/ CuIn_0.75_Ga_0.25_S_2_/Au	Spin‐coating	0.915	21.50	58.10	11.43	[177]
CuIn_0.75_Ga_0.25_S_2_ + 10% Spiro		FTO/c‐TiO_2_/m‐TiO_2_/Cs_0.05_FA_0.81_MA_0.14._PbI_2.55_Br_0.45_/ CuIn_0.75_Ga_0.25_S_2_ + 10% Spiro/Au	Spin‐coating	1.108	24.09	70.14	18.72	[177]
Aged CuIn_0.75_Ga_0.25_S_2_	(FA, MA)Pb(I, Br, Cl)_3_	ITO/SnO_2_/(FA, MA)Pb(I, Br, Cl)_3_/ CuIn_0.75_Ga_0.25_S_2_/C	Spin‐coating	0.989	24.1	54	12.9	[185]
Cu_12_Sb_4_S_13_ QDs	CH_3_NH_3_PbI_3_	ITO/TiO_2_/CH_3_NH_3_PbI_3_/ Cu_12_Sb_4_S_13_ QDs/Au	Spin‐coating	1.05	21.85	61.6	14.13	[186]
PVP/CZTS	CH_3_NH_3_PbI_3_	FTO/TiO_2_/ CH_3_NH_3_PbI_3_/CZTS/Au	Spin‐coating	0.99	18.62	60.3	11.17	[187]
CuIn_0.75_Ga_0.25_S_2_	CsMAFAPb(I, Br)_3_	FTO/c‐TiO_2_/mp‐TiO2/CsMAFAPb(I, Br)_3_/ CuIn_0.75_Ga_0.25_S_2_/C	Spin‐coating	1.10	23.18	65	16.45	[188]
NiOx/CuSCN	Cs_0.05_FA_0.81_MA_0.14_PbI_2.55_Br_0.45_	FTO/mp‐TiO_2_/Cs0_.05_FA_0.81_MA_0.14_PbI_2.55_Br_0.45_/NiOx/CuSCN/Au	Spin‐coating	1.030	22.10	72.82	16.58	[123]
Cs:NiOx/CuSCN		FTO/mp‐TiO_2_/Cs0_.05_FA_0.81_MA_0.14_PbI_2.55_Br_0.45_/Cs:NiOx/CuSCN/Au	Spin‐coating	1.086	23.01	73.73	18.42	[123]
Spiro‐OMeTAD/SnS_1‐x_O_2x_	Cs_0.05_(MA_0.15_FA_0.85_)_0.95_Pb(I_0.85_Br_0.15_)_3_(1.4M): CNT:TiO_2_	ITO/SnO_2_/Al_2_O_3_/ Cs_0.05_(MA_0.15_FA_0.85_)_0.95_Pb(I_0.85_Br_0.15_)_3_(1.4M): CNT:TiO_2_/Spiro‐OMeTAD/SnS_1‐x_O_2x_/Au	Spin‐coating	1.23	25.50	78	24.5	[179]
FBT‐Th_4_/Cu_x_O	CH_3_NH_3_PbI_3_	FTO/SnO2/PC60BM/ CH_3_NH_3_PbI_3_/ HTMs/Au	Spin‐coating	1.12	22.35	75.4	18.85	[189]
Cu(TFSI)_2_‐doped spiro‐OMeTAD	CH_3_NH_3_PbI_3_	FTO/ c‐TiO/PCBA/CH_3_NH_3_PbI_3_/ Cu(TFSI)_2_‐doped spiro‐OMeTAD/Ag	Evaporation	1.00	20.79	64.47	13.4	[190]
WSe_2_	CH_3_NH_3_PbI_3_	FTO/c‐TiO_2_/m‐TiO_2_/ CH_3_NH_3_PbI_3_/WSe_2_/Au	Spin‐coating	0.88	20.38	51.17	9.18	[191]
TiO_2_:N_b_ (Bandgap 3.0 eV)	FAPbI_3_	FTO/TiO_2_/FAPbI_3_/TiO2:N_b_/Ag	SCAPS‐1D simulation	1.25	21.760	83.44	22.83	[192]
Sb_2_S_3_	FAMAPb(IBr)_3_	FTO/c‐TiO_2_/m‐TiO_2_/FAMAPb(IBr)_3_/ Sb_2_S_3_/Au	Spin‐coating	0.98	14.04	60	8.2	[178]
Cu_3_SbS_4_	FAMAPb(IBr)_3_	FTO/c‐TiO_2_/m‐TiO_2_/FAMAPb(IBr)_3_/ Cu_3_SbS_4_/Au	Spin‐coating	1.015	18.84	68	13	[178]
MXene/Spiro‐OMeTAD	(4F‐PEA)_2_MA_4_Pb_5_I_16_	FTO glass/c‐TiO_2_/m‐TiO_2_/(4F‐PEA)_2_MA_4_Pb_5_I_16_/MXene/Spiro‐OMeTAD/Au	Spin‐coating	1.03	14.9	45	6.9	[193]
Spiro‐OMeTAD/AgI	PVSK	ITO/SnO_2_/PVSK/Spiro‐OMeTAD/AgI/Ag	Spin‐coating/Drop casting	1.08	24.18	73.94	19.31	[194]
FeS_2_	PVSK	FTO/c‐TiO_2_/m‐TiO_2_/PVSK/FeS_2_/Au	Spin‐coating	0.935	17.72	67.7	11.22	[195]
Spiro‐OMeTAD: MoS_2_/MoO_3_	CH_3_NH_3_PbI_3_	FTO/TiO_2_/CH_3_NH_3_PbI_3_/Spiro‐OMeTAD: MoS_2_/MoO_3_/Ag	Spin‐coating	1.10	20.18	75	19.88	[180]
PDCBT/Ta‐WO*_x_*	MA*_x_* GA_1‐_ *_x_* PbI_3_	ITO/SnO_2_/MA*_x_* GA_1‐_ *_x_* PbI_3_/PDCBT/Ta‐WO*_x_*/Carbon	Spin‐coating	1.127	22.5	63.7	16.2	[181]
P3HT/Ta‐WO*_x_*		ITO/SnO_2_/MA*_x_* GA_1‐_ *_x_* PbI_3_/P3HT/Ta‐WO*_x_*/Au	Spin‐coating	1.04	23.6	78.6	19.4	[181]
MnS	MAFAPb(I, Br)_3_	FTO/TiO_2_/ MAFAPb(I, Br)_3_/MnS/Au	Thermal Evaporation	1.11	23.4	77	19.9	[196]
TiS_2_	MAFAPb(I, Br)_3_	FTO/c‐TiO_2_/mp‐TiO_2_/ MAFAPb(I, Br)_3_/TiS_2_/Au	Spin‐coating	0.954	21.79	65	13.54	[197]
Spiro‐OMeTAD/MoO_x_	CH_3_NH_3_PbI_3_	FTO/c‐TiO_2_/mp‐TiO_2_/CH_3_NH_3_PbI_3_/Spiro‐OMeTAD/MoO_x_/Al	Spin‐coating	0.94	18.5	67	11.6	[198]

Transition metal dichalcogenides (e.g., WSe_2_, TiS_2_) and sulfides (e.g., MnS, FeS_2_) also emerged as capable HTLs, with MnS reaching a competitive 19.9% efficiency. Meanwhile, advanced doped and hybrid HTLs like spiro‐OMeTAD/SnS_1__−__x_O_2__x_ and spiro‐OMeTAD:MoS_2_/MoO_3_ demonstrated the highest efficiencies of 24.5% and 19.88%, respectively, benefiting from enhanced charge transport and suppressed recombination [179, 180]. Finally, polymer‐inorganic hybrids including P3HT/Ta‐WO_x_ offered good performance (∼17%–19%), with the latter providing improved thermal stability [181]. Overall, the landscape of inorganic HTLs in PSCs reveals substantial progress in efficiency and stability, with many alternatives approaching surpassing the performance of organic counterparts. Moving forward, the focus must remain on interface engineering, scalable deposition methods, and long‐term operational stability to transition these materials into commercial applications.

Table 5 summarize the photovoltaic performance of chalcogenide, sulphide and hybrid HTLs. Based on the type of HTL, the V_oc_ and FF significantly vary due to the energy alignment, charge transport, interfacial and hybridization strategies. For instance, GO as a single‐layer HTL shows significantly lower FF and V_oc._ The reason includes poor charge extraction and high series resistance, however, the use of carbon as the counter electrode make them cost‐effective [174]. In contrast, chalcogenide systems such as Cu(In,Ga)S_2_ show improved V_OC_ and FF due to tunable bandgap and better energy level alignment, enabling more efficient hole extraction. Furthermore, hybrid (organics plus inorganics) and doped HTLs (e.g., CuIn_0_._75_Ga_0_._25_S_2_ + Spiro, Spiro‐OMeTAD/SnS_1__−__x_O_2__x_, and MoS_2_/MoO_3_ composites) demonstrate significantly enhanced performance, with FF values exceeding 75% and PCEs above 20%, attributed to synergistic effects such as improved conductivity, reduced recombination, and enhanced interface passivation [179].

## 4 Emerging Inorganic HTLs

In addition to the widely studied inorganic HTLs discussed above, several emerging materials, including CuAlO_2_, CuScO_2_, CuBi_2_O_4_, SrCu_2_O_2,_ V_2_O_3_, MnO_x_, p‐type SnO_2_ have recently attracted attention [199, 200]. The delafossite oxides, including CuAlO_2_, CuScO_2_, CuBi_2_O_4_ offers high transparency and intrinsic p‐type conductivity. Similarly, V_2_O_3_, MnO_x_, Mn‐doped NiO_x_ and p‐type SnO_2_ provide tunable electronic structures and excellent processibility with the current device architecture [201]. However, most of these materials are currently being studied for p‐i‐n PSCs and are in early stages of device development and are limited to simulation using SCAPS‐1D [202]. As such, further investigation is required to fully assess the potential of these materials as scalable HTLs for n‐i‐p PSCs.

## 5 Stability of Encapsulated and Unencapsulated n‐i‐p PSCs Using Inorganic HTLs

A critical limitation in many reports using inorganic HTLs for n‐i‐p perovskite solar cells includes considering enhanced stability without encapsulation and fully adhering to the standardized International Summit on Organic Photovoltaics Stability (ISOS) protocols. These protocols include the standardized stability assessment of perovskite solar cells under encapsulation with reported metrics including T_80_ and T_S80_ [203].

For unencapsulated devices, the improvement in the stability for the n‐i‐p perovskite solar cells with inorganic HTL shows only the preliminary shelf life if the devices statistics, temperature, humidity and maximum power point tracking are not considered. The devices, unless encapsulated, can degrade differently under light and different environment factors due to HTL/perovskite interface for ion migration and interfacial redox reaction as well as electrode instability [204].

For encapsulated devices, stability depends on the combined effect of the inorganic HTL/perovskite interface and the encapsulation barrier. With encapsulation, the exposure to moisture and oxygen is reduced resulting in high retaining PCE. Encapsulation testing provides practical device durability but limits the intrinsic stability contribution of the HTL and HTL/perovskite interface for stability improvement [205].

Accordingly, stability comparison in this review is mainly based on the short‐term ambient storage (preliminary unencapsulated shelf life). A comparative review on the encapsulated and unencapsulated devices may be important as strong evidence to the robustness and state of commercialization of inorganic HTL based n‐i‐p PSCs.

## 6 Scalability and Commercial Viability of Inorganic HTLs

The deployment of perovskite solar cells with commercial viability requires high power conversion efficiency, stability, scalable manufacturing processes and cost‐effective materials. Inorganic hole transport materials, including Ni and Cu based components, can benefit from a well‐established global supply chain and high elemental abundance, providing them advantage over the complex synthesis route of organic HTLs.

For a current perovskite solar module (PCM), the material cost contributes to around 70% and the capital cost and other cost account for the rest of 30% [206]. The complete manufacturing cost for 100 MW PCM includes 0.391 $W^−1^ materials cost, 0.092 $W^−1^ capital cost (Including deposition equipment) and 0.088 $W^−1^ (including electricity, labor and maintained) [206]. The material cost in 2024 for a PCM was estimated as 29.3 $m^−2^ among which NiO_x_ only contribute to 0.356 $m^−2^ [206].

In contrast, HTMs such as spiro‐OMeTAD (organic HTL) contribute significantly higher material costs. For example, the top Au electrodes significantly contribute to the cost of the device. Due to its suitable work function and chemical stability, Au has significantly dominated the highly efficient laboratory‐based PSCs. However, the high cost of Au as a top electrode, may undermine the economic advantage offered by inorganic HTLs. Increasing research efforts to focus on replacing the high‐cost top electrodes with low‐cost electrodes, including carbon‐based electrodes and transparent electrodes is underway. Notably, several studies (as summarized in Tables 1, 2, 3, 4, 5) have demonstrated NiO_x_ and Cu‐based devices employing low‐cost electrodes, including Al and Carbon, highlighting viable pathways toward reducing reliance on Au [207].

In addition to the material costs, the choice of manufacturability is an important factor while considering the scalability of the PSCs using inorganic materials as HTL. Various processing methods, including sputtering, spray pyrolysis, sol‐gel and spin‐coating, have the scalability potential. However, the choice of deposition introduces a trade‐off between film quality and control, processing time, and throughput. High vacuum techniques provide highly uniform and reproducible films and includes the highest share in capital cost for the PCM (55% of total capital cost. However, it contributes less than 10% of the total manufacturing cost of the PCM, making it a sustainable technique for the fabrication of large‐scale PCM [206, 208].

Overall, while inorganic HTLs offer significant advantages over material stability and abundance, the commercial viability is linked to the development of a scalable and low‐cost device stack. Future research should not only focus on opto‐electronic properties of these materials but also optimizing them with industrially relevant fabrication processes and economically viable electrode systems.

## 7 Summary

In summary, this review critically examines inorganic hole transport materials (HTLs) for n‐i‐p perovskite solar cells. This includes nickel oxide (NiO_x_), copper‐based oxides, transition metals, and inorganic/organic composites. By comparatively analysing the photovoltaic performance, stability and interfacial energy levels, this review emphasizes the role of band alignment, charge transport and deposition process for the inorganic HTLs in n‐i‐p perovskite solar cells. The article briefly discussed emerging hole transport materials with limited work to simulation for n‐i‐p PSCs.

The exploration of inorganic hole transport layers in PSCs has revealed their significant potential to enhance both performance and stability. With ongoing advancements in material engineering, interface optimization, and scalable deposition techniques, these HTLs are steadily overcoming existing limitations, providing a pathway to improve the PSC efficiency and stability. The deposition techniques for the HTLs are dominated by solution processing methods, but vacuum deposition techniques with controlled film quality can bring innovative research for improved device efficiency and stability. As the field progresses, the continued integration of novel inorganic materials and their synergistic combinations with organic material pave the way for highly efficient, durable, and commercially viable perovskite solar technologies for building‐integrated and tandem solar cells.

## 8 Outlooks and Conclusion

In n‐i‐p PSCs, inorganic hole transport layers (HTLs) are considered as an excellent alternative to its organic counterparts in PSCs. In this architecture, inorganic HTLs provide benefits including high transmittance, superior chemical and thermal stability, tuneable electronic and electrical properties, better resistance to moisture and lower cost (as compared to organic materials). Materials such as NiO_x_, Cu‐based oxides, and transition multifunctional metal oxides, including VO_x_, CoO_x_, and CrO_x_ have demonstrated strong compatibility in n‐i‐p structure by facilitating efficient hole extraction materials. The enhanced hole extraction engineering by band energy level, conductivity, and interfacial quality alignment using suitable deposition methods. This includes doping strategies, interfacial engineering, and hybrid systems that combine inorganic HTLs with organic molecules such as Spiro‐OMeTAD to achieve higher power conversion efficiencies (PCEs) with improved stability. Despite all the progress, the assessment of device stability remains inconsistent across the literature as many studies rely on short‐term and non‐standardized testing conditions (ISOS guidelines), highlighting the requirement of more rigorous and comparable evaluation protocols.

Within the n‐i‐p configuration, several challenges remain to be addressed before inorganic HTLs can be fully used for large‐scale commercialization including the scalability and stability. Interface‐related defects and the chemical deposition techniques still hinder reproducibility and long‐term operational stability. Addressing these issues will require innovative approaches such as low‐temperature, vacuum‐based deposition, as well as surface passivation strategies to minimize trap‐assisted recombination. Additionally, doping and compositional engineering are expected to play a pivotal role in overcoming conductivity limitations, while scalable methods like sputtering, atomic layer deposition (ALD) and e‐beam evaporation can help improve uniformity and reproducibility. These vacuum‐based deposition methods, however requires further optimization to minimize plasma‐induced damage for the perovskite layer and production cost. Establishing a scalable hybrid process including both solution and vacuum‐based deposition of inorganic HTLs represents an important research gap toward the stable perovskite module commercialization in n‐i‐p PSCs.

Looking forward, the integration of inorganic HTLs for n‐i‐p PSCs into tandem architectures—particularly perovskite/silicon tandems—represents an exciting direction, as their transparency and durability can directly impact current matching and long‐term reliability. Furthermore, exploring ternary metal oxide HTLs for n‐i‐p architecture that provide transport for holes and also provide moisture resistance, UV shielding, or defect passivation may accelerate their application in stable device platforms. Similarly, combining the inherent robustness of inorganic materials with the tunability of organic and hybrid counterparts could lead to a new generation of HTLs for n‐i‐p PSC that balance stability, efficiency, and processability.

Overall, while inorganic HTLs for the n‐i‐p PSC architecture have demonstrated strong potential in terms of stability, environmental robustness and cost effectiveness, the highest certified power conversion efficiency is still predominantly achieved using organic HTLs such as spiro‐OMeTAD and PTAA. With inorganic HTLs showing competitive performance and stability, optimization is still required to fully bridge the efficiency gap. With continued efforts in interface engineering, doping optimization, systematic stability evaluation, hybridization strategies, and scalable fabrication, inorganic HTLs are poised to play a central role in advancing perovskite photovoltaics from the laboratory to commercial deployment, bridging the gap between record efficiencies and real‐world stability.

## Conflicts of Interest

The authors declare no conflicts of interest.

## Data Availability

The data that support the findings of this study are available from the corresponding author upon reasonable request.
